# *De novo* Assembly of the *Camellia nitidissima* Transcriptome Reveals Key Genes of Flower Pigment Biosynthesis

**DOI:** 10.3389/fpls.2017.01545

**Published:** 2017-09-07

**Authors:** Xingwen Zhou, Jiyuan Li, Yulin Zhu, Sui Ni, Jinling Chen, Xiaojuan Feng, Yunfeng Zhang, Shuangquan Li, Hongguang Zhu, Yuanguang Wen

**Affiliations:** ^1^Guangxi Key Laboratory of Forest Ecology and Conservation, College of Forestry, Guangxi University Nanning, China; ^2^College of Biology and Pharmacy, Yulin Normal University Yulin, China; ^3^Research Institute of Subtropical Forestry, Chinese Academy of Forestry Fuyang, China; ^4^College of Marine Sciences, Ningbo University Ningbo, China

**Keywords:** RNA-sequencing, transcriptome, metabolic pathway, carotenoids, flavonoids, flower color, *Camellia nitidissima*

## Abstract

The golden camellia, *Camellia nitidissima* Chi., is a well-known ornamental plant that is known as “the queen of camellias” because of its golden yellow flowers. The principal pigments in the flowers are carotenoids and flavonol glycosides. Understanding the biosynthesis of the golden color and its regulation is important in camellia breeding. To obtain a comprehensive understanding of flower development in *C. nitidissima*, a number of cDNA libraries were independently constructed during flower development. Using the Illumina Hiseq2500 platform, approximately 71.8 million raw reads (about 10.8 gigabase pairs) were obtained and assembled into 583,194 transcripts and 466, 594 unigenes. A differentially expressed genes (DEGs) and co-expression network was constructed to identify unigenes correlated with flower color. The analysis of DEGs and co-expressed network involved in the carotenoid pathway indicated that the biosynthesis of carotenoids is regulated mainly at the transcript level and that *phytoene synthase* (*PSY*), β *-carotene 3-hydroxylase* (*CrtZ*), and *capsanthin synthase* (*CCS1*) exert synergistic effects in carotenoid biosynthesis. The analysis of DEGs and co-expressed network involved in the flavonoid pathway indicated that *chalcone synthase* (*CHS*), *naringenin 3-dioxygenase* (*F3H), leucoanthocyanidin dioxygenase(ANS)*, and *flavonol synthase* (*FLS*) play critical roles in regulating the formation of flavonols and anthocyanidin. Based on the gene expression analysis of the carotenoid and flavonoid pathways, and determinations of the pigments, we speculate that the high expression of *PSY* and *CrtZ* ensures the production of adequate levels of carotenoids, while the expression of *CHS, FLS* ensures the production of flavonols. The golden yellow color is then the result of the accumulation of carotenoids and flavonol glucosides in the petals. This study of the mechanism of color formation in golden camellia points the way to breeding strategies that exploit gene technology approaches to increase the content of carotenoids and flavonol glucosides and to decrease anthocyanidin synthesis.

## Introduction

The camellia, a traditional and much-loved flower in China, is a well-known and popular ornamental plant around the world. The flowers of most camellia species and cultivars are red, pink, white or purple, and yellow flowers are rare. The breeding of yellow camellias of high ornamental value, such as cultivars with large flowers or that are polypetalous, is an important goal for plant breeders and for research into the breeding of ornamentals.

The golden camellia, *Camellia nitidissima* Chi., known as “the queen of camellias” or “dreaming camellia” because of its golden yellow flowers, Chang and Ren (1998) and Gao (2005) is a highly-prized plant that is found naturally in the Guangxi Zhuang Autonomous Region of China. Since it was first discovered in the 1960s, this rare and unique genetic resource has been used in attempts to breed yellow camellia cultivars (Chen, 1987). However, despite almost half a century of effort using traditional breeding methods, generations of hybrid progeny of the golden camellia do not display the expected yellow flower color (Zhao et al., 1998; Parks, 2000; Nishimoto et al., 2004). One reason for the slow progress in the use of the golden camellia in hybrid breeding is that the biosynthesis of the golden yellow color and its regulation remain unclear, and as a result informed breeding programs cannot be pursued.

Analyses have shown that the golden yellow petals of *C. nitidissima* contain a number of flavonoid and carotenoid pigments, such as quercetin-7-*O*-glycoside (Qu7G), quercetin-3-*O*-glycoside (Qu3G), quercetin, xanthophyll, and neoxanthin (Miyajima et al., 1985; Parks and Scogin, 1987; Peng et al., 2011; Zhou, 2012). Work has indicated that although carotenoids may contribute more to the yellow color than flavonoids, cooperative effects of the two classes may increase the degree of yellow coloration (Parks and Scogin, 1987; Zhou, 2012). The flavonoid biosynthetic pathway has been comprehensively characterized, and the associated genes have been identified in many plants, in numerous studies (Winkel-Shirley, 2001; Tanaka et al., 2008; Falcone Ferreyra et al., 2012). In *C. nitidissima*, only a few of the genes involved in flavonoid pigment biosynthesis, notably genes encoding chalcone synthase (Zhou et al., 2011), chalcone isomerase (Zhou et al., 2012), flavanone 3-hydroxylase (Zhou et al., 2015), and flavonol synthase (Zhou et al., 2013), have been cloned and studied, and many other genes and the associated regulatory mechanisms remain uncharacterized. Similarly, although carotenoid pigments are conspicuous in the flowers (or fruits) of a range of plant species, and the carotenoid biosynthesis pathway in plants has been extensively studied (Tanaka et al., 2008; Cazzonelli and Pogson, 2010; Giuliano, 2014), few of the carotenoid biosynthesis genes of *C. nitidissima* have been cloned. Many structural genes, including multiple copies, remain to be characterized in this species, together with the regulatory mechanisms.

In this study, we used RNAseq to comprehensively profile the mRNA populations present at different developmental stages of the golden camellia flower. Critical changes were found in the expression of genes involved in carotenoid and flavonoid pigment biosynthesis. In addition, we identified modules of genes related to carotenoid and flavonoid biosynthesis that were co-expressed during the flower development process, and further explored hub genes and important gene interactions in these modules. The findings provide a systems-level context for further studies on the multi-gene regulation of carotenoid and flavonoid biosynthesis in *C. nitidissima*.

## Materials and methods

### RNA sequencing library construction and sequencing

#### RNA preparation

**Figure 1:**
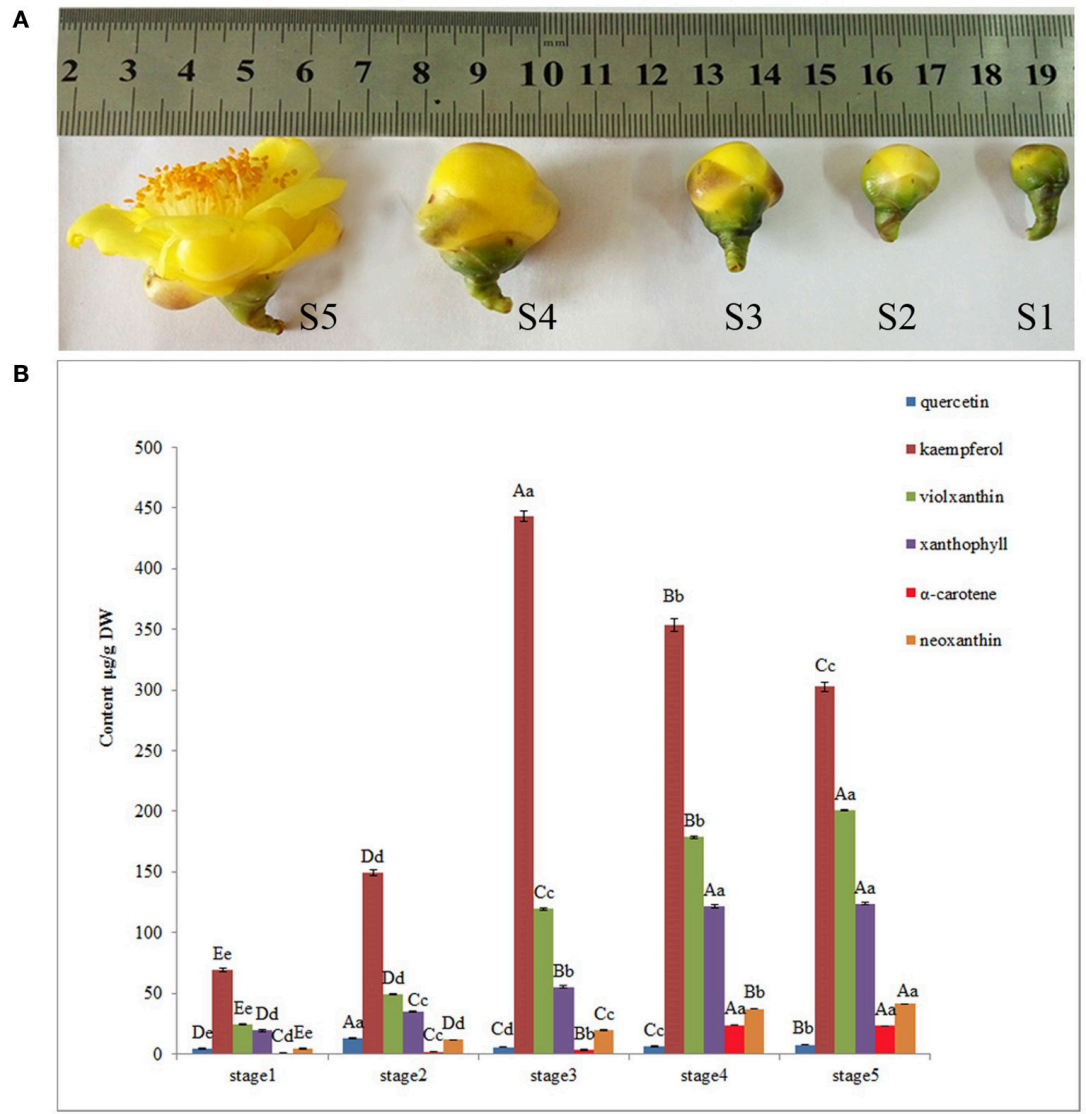
Pigment analysis during floral development stages of *Camellia nitidissima*. **(A)**, Different stages of floral buds: S1–S5 indicate the five developmental stages; buds average diameter: S1, 0.8 ± 0.2 centimeter; S2, 1.2 ± 0.2 centimeter; S3, 1.8 ± 0.2 centimeter; S4, 2.6 ± 0.3 centimeter; S5, 4.5 ± 0.5 centimeter. **(B)**, HPLC determination of pigments, including quercetin, kaempferol, neoxanthin, violaxanthin, xanthophyll and α–carotene. Significant differences (*P* < 0.05) among different samples were performed by SPSS17.0 software and shown in different upper case letters.

Plants of *Camellia nitidissima* Chi. were grown in the garden of Yulin Normal University (Yulin city, Guangxi, China). The mean temperature was about 21°C. Healthy floral buds at five different developmental stages of *C. nitidissima*, denoted Stages 1-5 (S1-S5), were collected (Figure 1A). Total RNA from each of 15 flower samples (i.e., three biological replicates from different plants for each developmental stage) was extracted using a modified CTAB (cetyltrimethylammonium bromide) method (Gasic et al., 2004). Samples were snap-frozen in liquid nitrogen and stored at −70°C prior to processing. RNA integrity was confirmed using an Agilent 2100 Bioanalyzer (Agilent Technologies, Santa Clara, CA), setting a minimum integrity number value of 8.

#### Preparation of strand-specific cDNA library for transcriptome sequencing

For each sample, 5 μg of total RNA was used to isolate mRNA for the preparation of a strand-specific RNA-seq library, using a NEBNext Poly (A) mRNA Magnetic Isolation Module and NEBNext Ultra Directional RNA Library Prep Kit for Illumina (New England Biolabs, Ipswich, MA, USA) and following the manufacturer's protocols, with minor modifications. Briefly, mRNA was extracted with 15 μl of NEBNext Magnetic Oligo d(T)25 and fragmented in NEBNext First Strand Synthesis Buffer by heating at 94°C for 10 min. First-strand cDNA was reverse-transcribed from the fragmented mRNA and then used as a template to synthesize double-stranded cDNA, with dUTP replacing dTTP. The resulting double-stranded cDNA was end-repaired, dA-tailed and then ligated with NEBNext Adaptor. To remove unwanted large fragments, the adaptor-ligated cDNA was selected for size using Agencourt AMPure XP beads (Beckman Coulter, Pasadena, CA, USA) in 0.6 volumes of the ligation reaction. For optimizing the size selection, another round of size selection was performed, as described by Zhong et al. (2011), in which the beads were used in 1.4 volumes of the cDNA solution. Next, the selected cDNA was digested with NEBNext USER enzyme (New England Biolabs) and then amplified by PCR under the following conditions: 98°C for 30 s; 14 cycles of 98°C for 10 s, 65°C for 30 s, and 72°C for 30 s; 72°C for 5 min; and, finally, kept at 4°C. The PCR-amplified cDNA library was purified using 1.4 volumes of Agencourt AMPure XP beads and eluted in 20 μl of low TE buffer (10 mM Tris-HCl, pH 8.0, and 0.1 mM EDTA). Each purified library was then quantified by means of a Qubit 2.0 (Life Technologies, Carlsbad, CA, USA) fluorometer, using a dsDNA HS Assay Kit (Invitrogen/Life Technologies, Carlsbad, CA, USA).

#### Illumina sequencing

The cDNA library was sequenced from both the 5′ and 3′ ends on the Illumina Hiseq2500 platform (Illumina, San Diego, CA), according to the manufacturer's instructions. The fluorescent image process to sequences, base-calling and quality value (*Q*-value) calculation were performed by the Illumina data processing pipeline (version 1.4), in which 150-bp paired-end reads were obtained.

### *De novo* assembly of sequencing reads and sequence clustering

Before assembly, the raw reads were filtered to obtain high-quality clean reads by removing adaptor sequences, duplication sequences, reads containing more than 10% “N” rate (the “N” character representing ambiguous bases in reads), and low-quality reads containing more than 50% of bases with a *Q* ≤ 5.

Three commonly-used assembly pipelines, SOAPdenovo-trans (Xie et al., 2014), Bridger (Chang et al., 2015), and Trinity (Grabherr et al., 2011; Schulz et al., 2012) were compared and the result of different assemblers, including mapped reads and full transcript coverage, were evaluated. BUSCO software was used to conduct completeness analysis of the transcriptome (Simão et al., 2015). To reduce any sequence redundancy, the scaffolds were clustered using CD-hit (Li and Godzik, 2006). The clustering output was passed to CAP3 assembler (Huang and Madan, 1999) for multiple alignment and consensus building.

### Functional annotation and classification

The TransDecoder utility (Grabherr et al., 2011) was used to identify candidate coding regions within transcript sequences generated by *de novo* RNA-Seq transcript assembly using Trinity. Blast2GO software (Conesa et al., 2005) was used to conduct gene annotation. Conserved domains/families in the assembled unigenes were further identified using the InterPro database (version 30.0) (Hunter et al., 2009), the Pfam database (version 24.0) (Finn et al., 2010), and the Clusters of Orthologous Groups database at NCBI (Tatusov et al., 2001). Domain-based comparisons with the InterPro, Pfam, and COGs databases were performed using the InterProScan, HMMER3 and BLAST+ programs (*E*-value threshold: 10-5), respectively, with an *E*-value threshold of 10-5. KEGG pathways annotation was performed by sequence comparisons against the Kyoto Encyclopedia of Genes and Genomes database (Kanehisa, 2002), using the BLASTX algorithm (*E*-value threshold: 10-5).

### Transcript quantification and analysis of differentially expressed genes

The reads from the 15 libraries were aligned to the assembly transcriptome using Bowtie2 software (Langmead and Salzberg, 2012). Transcript quantification was performed using eXpress 1.5.0 software (Mangul et al., 2011). Based on the transcript and gene-level abundance estimates for each of the 15 samples, a matrix of counts and a matrix of normalized expression values were constructed using the scripts from the Trinity package. Analysis of differentially expressed genes (DEGs) was conducted using the edgeR package (Robinson et al., 2010).

### Gene validation and expression analysis

Five selected unigenes with potential roles in theanine and flavonoid synthesis were chosen for validation using real-time qPCR, with gene-specific primers designed using Primer Premier software (version 5.0) (Supplementary Table 1). One microgram of total RNA was used for reverse transcription in a total volume of 20 μL in the presence of 6-mer random primer and oligo primer, according to the Takara (Dalian, China) protocol. The standard curve for each gene was obtained by real-time PCR with several dilutions of cDNA. The reaction was performed in 20 μL, containing 10 μL of 2 × SYBR Green Master Mix (Takara, Dalian, China), 300 nM of each primer and 2 μL of 10-fold-diluted cDNA template. The PCR reactions were run in a Bio-Rad Sequence Detection System (Bio-Rad Laboratories, Hercules, CA, USA), using the following program: 95°C for 10 s; then 40 cycles of 95°C for 15 s followed by annealing at 60°C for 30 s. Subsequently, the specificity of the individual PCR amplification was checked using a heat dissociation protocol from 55° to 95°C following the final cycle of the PCR, and agarose gel electrophoresis. Triplicates of each reaction were performed, and the *18S rRNA* gene was chosen as an internal control for normalization following comparison of the expression of each of four reference genes (*actin, GAPDH, 18S rRNA*, and β*-tubulin*) at the development stages of S1-S5. Quantification of the relative expression of the genes was performed at three different stages using the delta-delta Ct method, as described by Livak and Schmittgen (2001). All data were expressed as the mean ± SD after normalization.

### Pigment analysis and weighted gene co-expression network analysis

#### Pigment analysis

The flavonoid pigments quercetin and kampferol were extracted and analyzed according to the method (Zhou, 2012). The epicatechin was extracted and analyzed according to the method (Huang et al., 2012 Carotenoid pigments were extracted from the freezed dried petals (about 50 mg) in 2 mL of acetone (90% v/v) for 3 h at 4°C in the dark (Tian et al., 2009). Following filtration of the extract through a 0.22-μm microporous membrane, high-performance liquid chromatography (HqPLC) analysis was carried out on an Inertsil ODS-SP (4.6 mm × 250 mm; 5 μm) column (GL Sciences, Japan) at a column temperature of 30°C using a LC-20AT instrument (Shimadzu, Kyoto, Japan), eluted at 0.8 mL/min with a linear gradient of *iso-*propanol (solvent A) and acetonitrile:H_2_O (80:20, v/v; solvent B), from 0% A and 100% B (at 0 min) to 100% A and 0% B (at 40 min). Neoxanthin, α-carotene, and xanthophyll were detected by measurement of absorbance at 450 nm and were quantified by comparison of elution times and peak areas with those of known standards chromatographed under the same conditions. Standards of neoxanthin, α-carotene, and xanthophyll for HPLC were obtained from Wako (Japan), quercetin, epicatechin and kampferol were from Sigma (Germany). The significant differences among different samples were analyzed using SPSS17.0 software.

#### Weighted gene co-expression network analysis

The R package WGCNA (Langfelder and Horvath, 2008) was used to identify modules of highly correlated genes, based on the normalized expression matrix data. The R package (Liu et al., 2010)was used to filter the genes based on genes expression and variance, in the end, 92106 genes were remained. With the help of the function pickSoftThreshold in the WGCNA package, the soft thresholding power was chosen as 21. The power was interpreted as a soft threshold of the correlation matrix. The resulting adjacency matrix was then converted to a topological overlap (TO) matrix by the TOM similarity algorithm. Genes were hierarchically clustered based on TO similarity. We used the Dynamic Hybrid Tree Cut algorithm 30 to cut the hierarchical clustering tree, and defined modules as branches from the tree-cutting. We summarized the expression profile of each module by representing it as the first principal component (referred to as module eigengene). Modules whose eigengenes were highly correlated (correlation above 0.8) were merged.

## Results

### Expression profile of the transcriptome in golden yellow petals in relation to flower development

**Figure 2:**
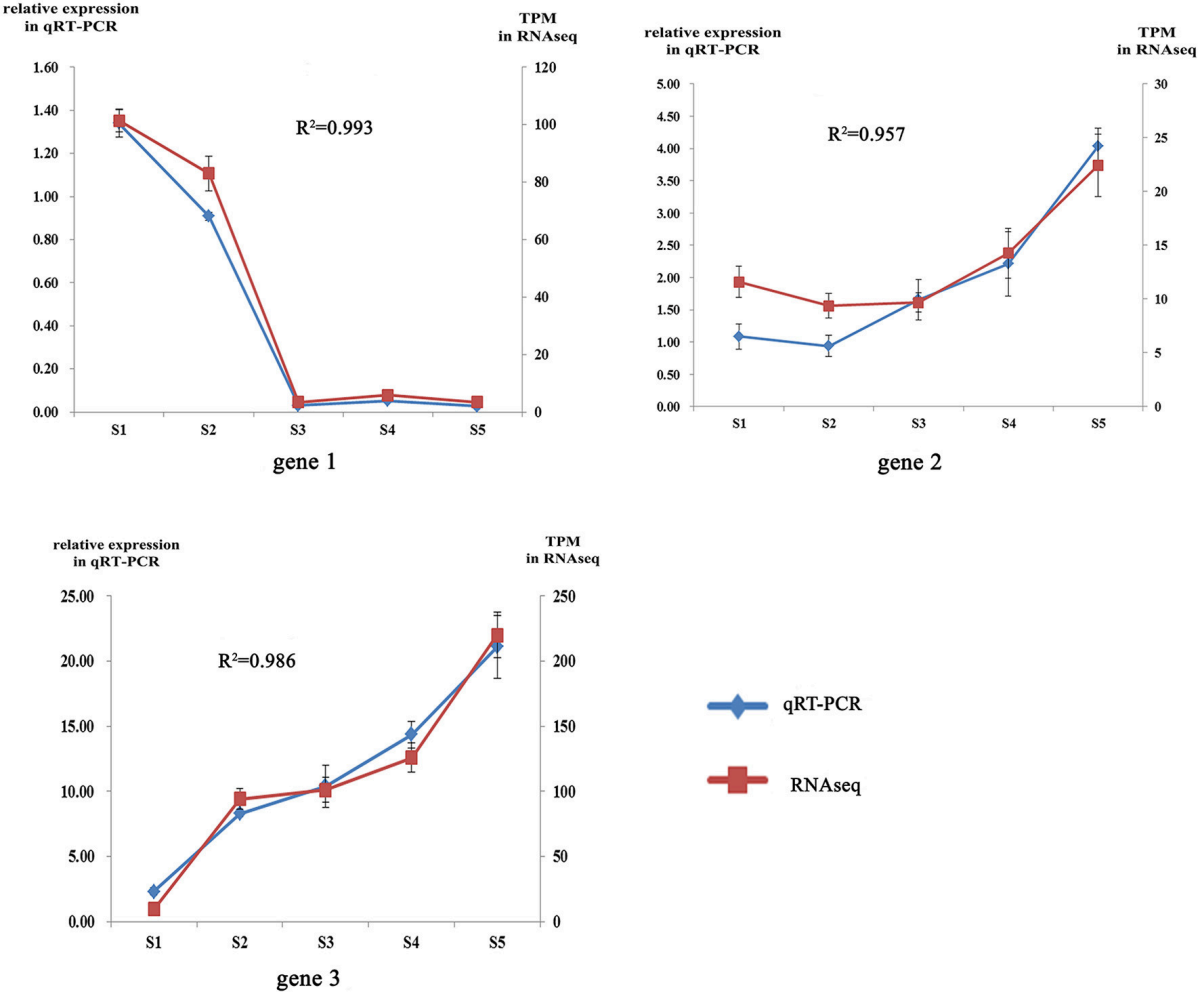
Correlation analysis of qRT-PCR and RNAseq.

To obtain an overview of the flower transcriptome of *C. nitidissima* at different developmental stages, RNA-seq strand-specific libraries from three biological replicates of *C. nitidissima* plants sampled at five developmental stages (15 libraries in total) were prepared for transcriptome analysis. In total, 71.8 million raw reads with a length of 150 bp (approximately 10.8 gigabase pairs (Gbp) of raw data) were obtained and the average Q30 was more than 93.55% (Supplementary Table 2). Three assemblers were compared with each other and the results showed that Trinity platform performed better in both count of full transcripts, remapped reads ratio and the completeness of transcriptome (Suplementary Tables 3, 4). The Trinity platform assembled 583,194 transcripts and 466,594 unigenes. Bench-marking universal single-copy orthologs (BUSCO) v.3 results using the embryophyta_odb9 gene set indicated that the transcriptomes have a moderate to high level of completeness (Suplementary Table 5). After transcript quantification of each biological replicate, correlation analysis suggested the biological replicates were well correlated (Supplementary Figure S1). To validate the TPM (transcripts per million) values as an accurate measure of transcript abundance, the expression levels of three representative genes were also assessed using qRT-PCR (Supplementary Table 6). The relative expression levels of these three genes (gene1, gene2, gene3) from qRT-PCR were closely correlated with their TPM values, with Pearson correlation coefficient values of 0.87, 0.82, and 0.802 at the significance level of *P* < 0.01(Figure 2). All raw high throughput sequence data have been deposited in the SRA database with accession number SRP112181.

Next, all the unigenes were annotated on the basis of sequence similarity, using four public databases: the National Center for Biotechnology Information (NCBI), the Non-redundant protein database (Nr), The Arabidopsis Information Resource (TAIR), the Swiss-Prot protein database (Swiss-Prot), and the Translated EMBL Nucleotide Sequence database (TrEMBL). In total, 237,538 (50.90%) unigenes were annotated, while the remaining 229,056 (49.09%) had no significant matches to any sequences in the public databases. Our work and the work of others has revealed numerous new genes specific to *C. nitidissima* that are of unknown function, which will be the subject of future studies.

We then assigned the unigenes to Gene Ontology (GO) terms for the categorization of standardized gene functions. A total of 36,146 unigenes could be allocated to three main categories: cellular component, biological process, and molecular function (Supplementary Figure S2). In the cellular component category, “the cell” and “cell part” corresponded to more than 20.0%, respectively. The major subgroupings within the biological process category included “metabolic process,” “cellular process,” “response to stimulus,” “pigmentation,” and “biological regulation.” The category of molecular function included “binding,” “catalytic activity,” “transporter activity,” and “structural molecule activity,” etc.

**Figure 3:**
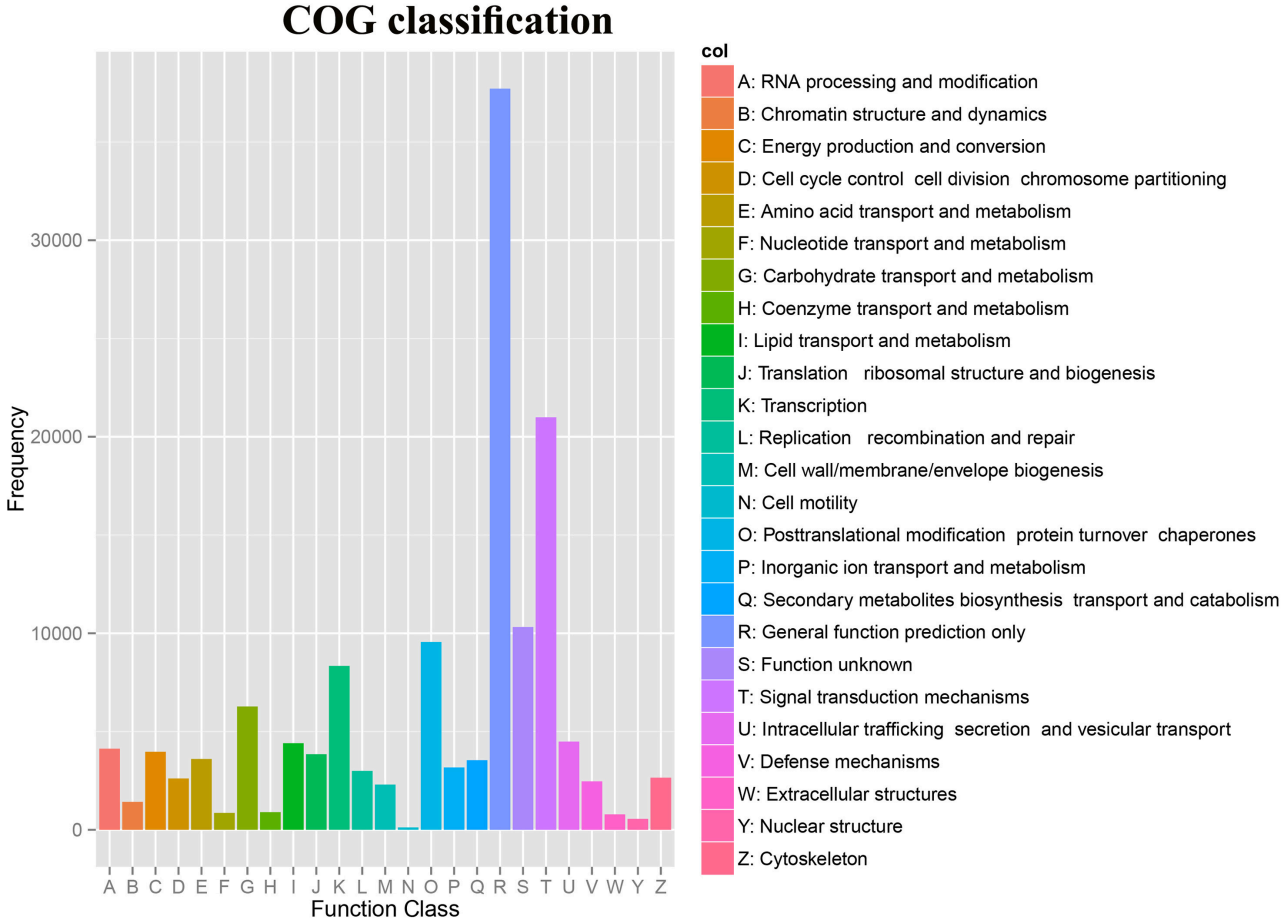
COG classification of unigenes from *Camellia nitidissima*.

The Clusters of Orthologous Groups (COG) database was used to annotate the assembled unigenes. Our study, a total of 142,016 unigenes were assigned to 24 COG clusters (Supplementary Table 7). The largest category was “General functions prediction only,” followed by “Signal transduction mechanisms,” “Function unknown,” “Posttranslational modification, protein turnover, chaperones,” “Transcription”, and “Carbohydrate transport and metabolism” (Figure 3).

**Figure 4:**
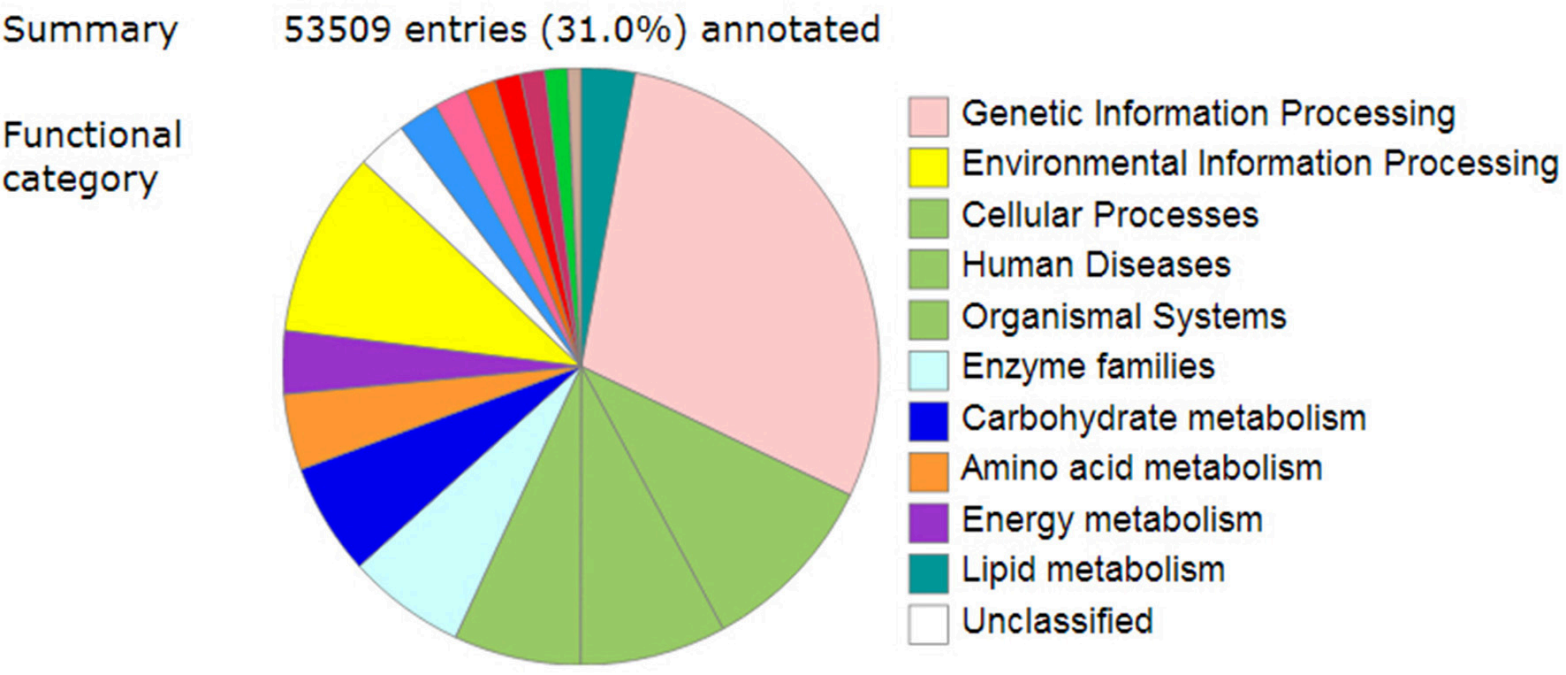
Overview of KEGG-annotated summary of unigenes from *Camellia nitidissima*.

To further determine the metabolic pathways involved in the pigment formation process, all the unigenes were searched against the KEGG (Kyoto Encyclopedia of Genes and Genomes) database and a total of 53,509 entries were annotated (Figure 4). The pathways that were most represented were “Genetic information processing,” followed by “Environmental information processing,” “Cellular Processes,” “Human diseases,” “Organismal systems,” “Enzyme families,” “Carbohydrate metabolism,” “Amino acid metabolism,” and “Energy metabolism” (Supplementary Table 8).

### Analysis of differentially expressed genes (DEGs) during flower development

**Figure 5:**
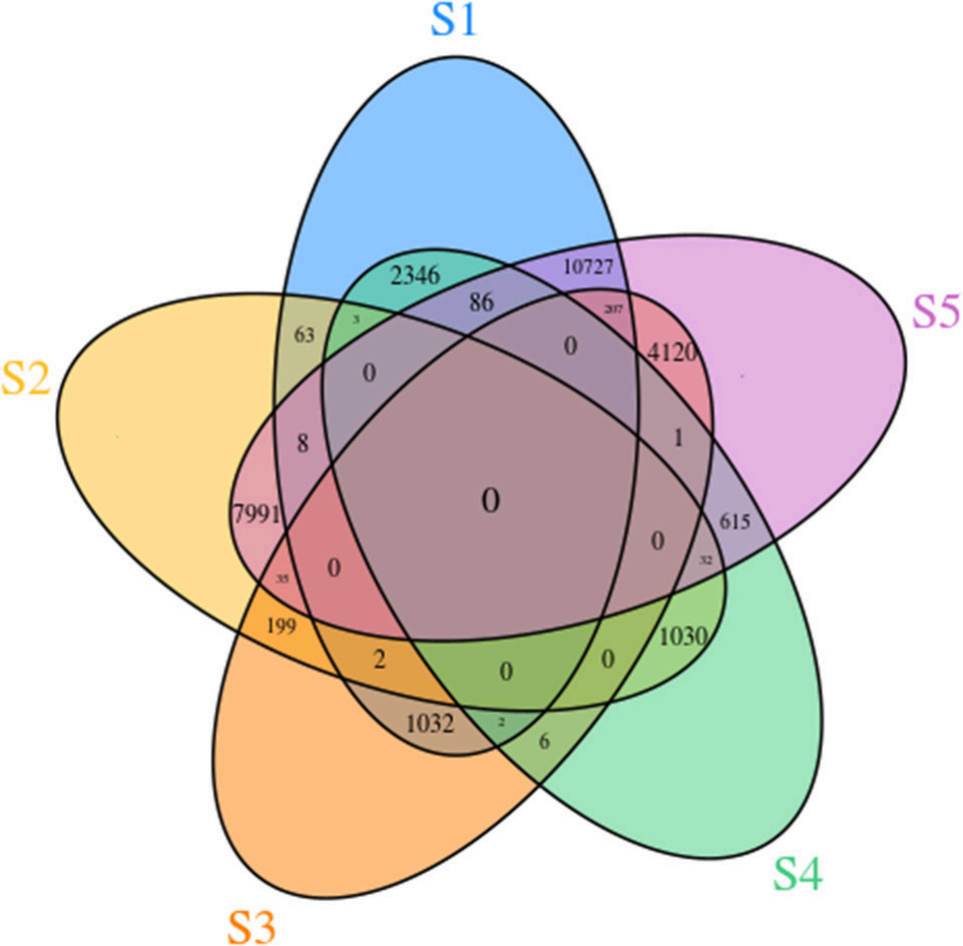
Venn diagram of unigenes involved in the five different flower developmental stages of *Camellia nitidissima*.

To identify DEGs during flower development, we compared transcript levels of each unigene between developmental stages. In total, 14,309 genes were differentially expressed at different stages. In the S2 vs. S1 comparison, 76 DEGs were detected, including 71 that were up-regulated and 5 that were down-regulated. In the S3 vs. S2 comparison, 58 up-regulated and 178 down-regulated transcripts were found. In the comparison of S4 vs. S3, 2 up-regulated and 7 down-regulated transcripts were detected. Finally, in the comparison of S5 vs. S4, 402 up-regulated and 332 down-regulated transcripts were revealed (Supplementary Table 9). All the pairwise comparisons and the overlaps between them were visualized in the form of a Venn diagram (Figure 5). This revealed that genes that showed changes in expression throughout all five developmental stages were rare.

### Co-expression network analysis based on flower pigments

**Figure 6:**
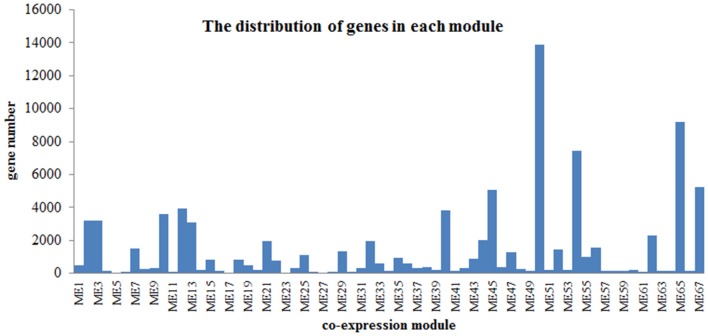
Distribution of genes in each module.

Flower pigment analysis by HPLC indicated that the highest content of quercetin occurred at S2, whereas that of kaempferol was at S3 (Figure 1B). Otherwise, variations in content for these two flavonols showed a similar trend. Levels of the four carotenoids violaxanthin, neoxanthin, xanthophyll, and α-carotene varied in concert, increasing from S1 to S5. To reveal the regulatory network correlated with the changes in the levels of the major pigments occurring during flower development, we associated the pigments with co-expression modules obtained by weighted gene co-expression network analysis (WGCNA)(Zhang and Horvath, 2005; Langfelder and Horvath, 2008). From WGCNA, 67 co-expression modules were constructed; of these, ME50 was the largest module, consisting of 13,898 genes, whilst ME17 was the smallest, consisting of only 35 genes. The distribution of unigenes in each module is shown in Figure 6.

**Figure 7:**
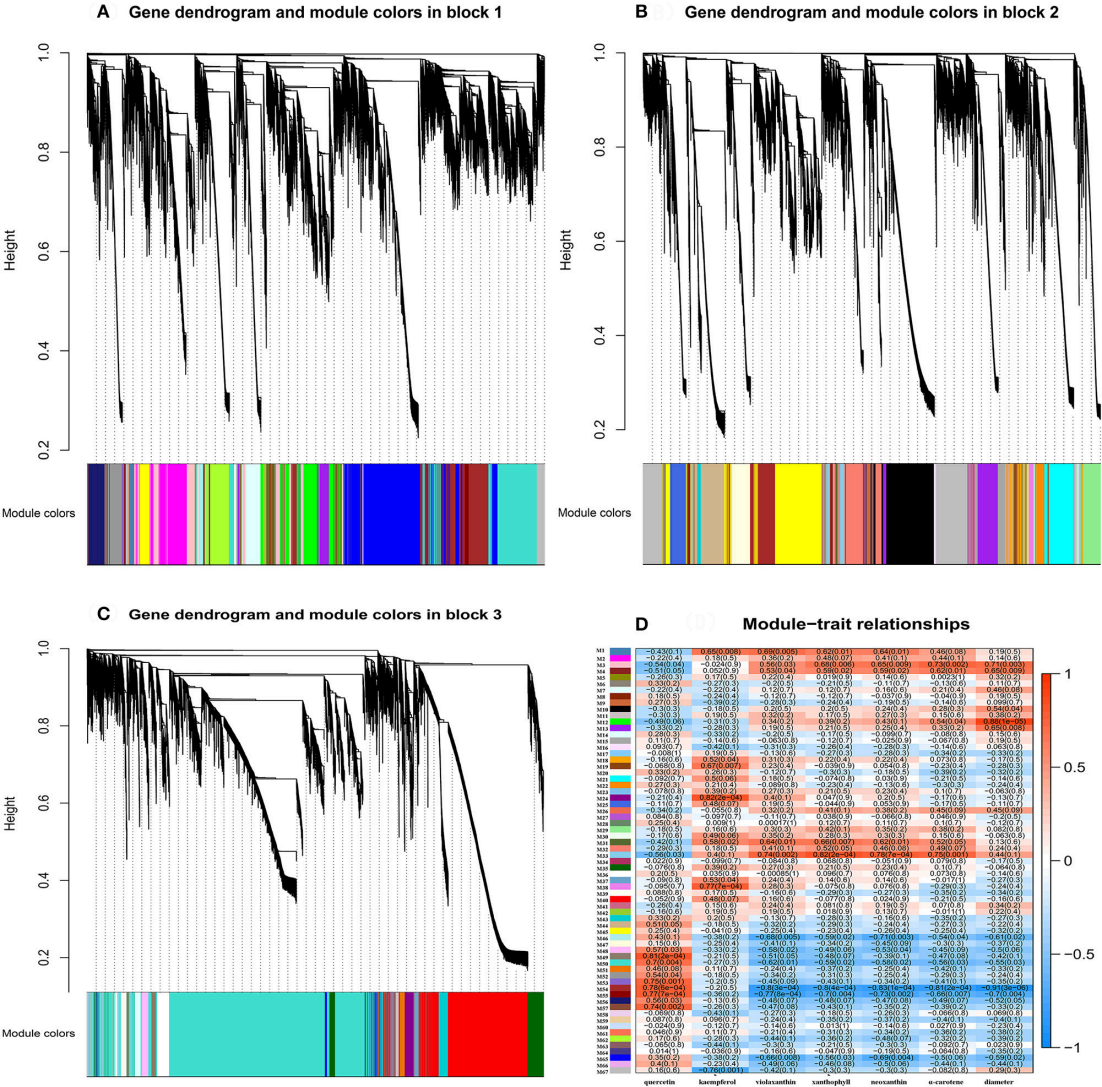
Gene co-expression modules detected using WGCNA. **(A)**, Clustering dendrogram of genes of block 1 across all the samples, with dissimilarity based on topological overlap, together with assigned merged module colors (merged dynamic) and the original module colors (dynamic tree cut). **(B)**, As for **(A)** above, but for genes of block 2. **(C)**, As for **(A)** above, but for genes of block 3. **(D)**, Module-trait associations. Each row corresponds to a module eigengene and each column to a trait. Each cell contains the corresponding correlation and *P*-value. The table is color-coded by correlation, according to the color legend.

Association of each co-expression module with each pigment was quantified by Pearson correlation coefficient (PCC) analysis and visualized using a hierarchically clustered heatmap (Figure 7). Modules of interest were selected according to the criteria |r| > 0.6 and *P* < 0.05. A number of modules displayed a close relationship with specific pigments, or with bud diameter, as follows (Supplementary Table 10): M1 – kaempferol, violaxanthin, xanthophyll and neoxanthin; M3 – xanthophyll, neoxanthin, α-carotene, and bud diameter; M4 – α-carotene and bud diameter; M31 – violaxanthin, xanthophyll, neoxanthin; M33 – violaxanthin, xanthophyll, neoxanthin, and α-carotene; M46 – violaxanthin and neoxanthin; M54 and M55 – quercetin, violaxanthin, xanthophyll, neoxanthin, α-carotene, and bud diameter; and M65 – violaxanthin and neoxanthin.

In contrast, other modules showed a specific relationship with bud diameter alone, or with a single pigment. Thus, M11 and M13 were correlated specifically with bud diameter, M19, M24, M38, and M67 were correlated specifically with kaempferol, and M49, M50, M53, and M57 showed a specific relationship with quercetin. The demonstration of these specific relationships is suggestive of the expression of particular genes associated with the biosynthesis or degradation of the substance concerned.

Those phenotype-related gene modules were further annotated by KEGG and GO analysis. In M54, flavonoid pathway was enriched (*P* < 0.01). The result showed that *chalcone synthase* (*CHS*, [EC:2.3.1.74, 2.3.1.170]), *naringenin 3-dioxygenase* (*F3H*, [EC:1.14.11.9]), *flavonol synthase* (*FLS*, [EC:1.14.11.23]) and *leucoanthocyanidin dioxygenase* (*LDOX/ANS*, [EC:1.14.11.19]) co-expressed (Supplementary Figure S3). It is known that CHS catalyzes the synthesis of chalcones, which are important in the flavonoid biosynthesis process. F3H catalyzes naringenin to yield dihydrokaempferol. F3H also catalyzes the hydroxylation of eryodictyol and pentahydroxyl flavanones to dihydroquercetin and dihydromyricetin, respectively. FLS catalyzes dihydroflavonols to flavonols, such as quercetin or kaempferol. LDOX/ANS catalyzes the synthesis of corresponding colored anthocyanidins. The result indicated that these genes may play important role in the biosynthesis of quercetin.

The co-expression gene modules related with α-carotene, violaxanthin, xanthophyll, neoxanthin were further explored (Figure 6, Supplementary Table 10). In M3, carotenoid pathway was enriched (*P* < 0.01). The result showed that *phytoene synthase*(*PSY*,[EC:2.5.1.32, 2.5.1.99]), ζ*-carotene isomerase*(*Z-ISO*, [EC:5.2.1.12]), *LUT5*: *beta-ring hydroxylase*; *zeaxanthin epoxidase*(*ZEP*, [EC:1.14.15.21]) and *9-cis-epoxycarotenoid dioxygenase* (*NECD*, [EC:1.13.11.51]) co-expressed (Supplementary Figure S4). In carotenoid biosynthesis, PSY is the first key enzyme which catalyzes geranylgeranylpyrophosphate (GGPP) to phytoene. Z-ISO catalyzes 9, 15, 9′- tri-cis-ζ-carotene to 9,9′-di-cis-ζ-carotene. LUT5 catalyzes the synthesis of zeinoxanthin or β -Cryptoxanthin. ZEP catalyzes zeaxanthin to violaxanthin. Both 9-cis-violaxanthin and 9′-cis-neoxanthin are cleaved to xanthoxin by NCED. These co-expressed genes may play important role in the biosynthesis of violaxanthin, xanthophyll, neoxanthin and α-carotene. Besides, there are no unigenes mapped to carotenoid pathway in M1, M46, M54, M55, and M65.

### Analysis of carotenoid pathway gene expression during flower development

In order to identify the structural genes involved carotenoid biosynthesis, all the unigenes were searched against the carotenoid biosynthesis pathway in the KEGG database. From this, a metabolic map was eventually compiled in which multiple transcripts encoding enzymes of the carotenoid biosynthesis pathway were displayed (Supplementary Figure S5). As indicated in the map, it is assumed that carotenoids are derived from geranyl- geranyl-PP and converted to neoxanthin or lutein by the action of the following enzymes: phytoene synthase (PSY, 7 unigenes), phytoene desaturase (PDS, 9 unigenes), ζ-carotene isomerase (Z-ISO, 2 unigenes), ζ-carotene desaturase (ZDS, 4 unigenes), carotenoid isomerase (CRTISO, 4 unigenes), lycopene β-cyclase (LCYB, 1 unigene), lycopene ε-cyclase (LCYE, 4 unigenes), β-carotene 3-hydroxylase (CrtZ, 3 unigenes), β-carotene hydroxylase (BCH, 10 unigenes), carotene ε-monooxygenase (LUT1, 4 unigenes), zeaxanthin epoxidase (ZEP, 8 unigenes), and neoxanthin synthase (NXS, 1 unigene). In addition to the unigenes assigned to the carotenoid biosynthetic pathway, many unigenes not involved in the biosynthesis of lutein or neoxanthin were detected. These genes may have important functions in other metabolic processes.

**Figure 8:**
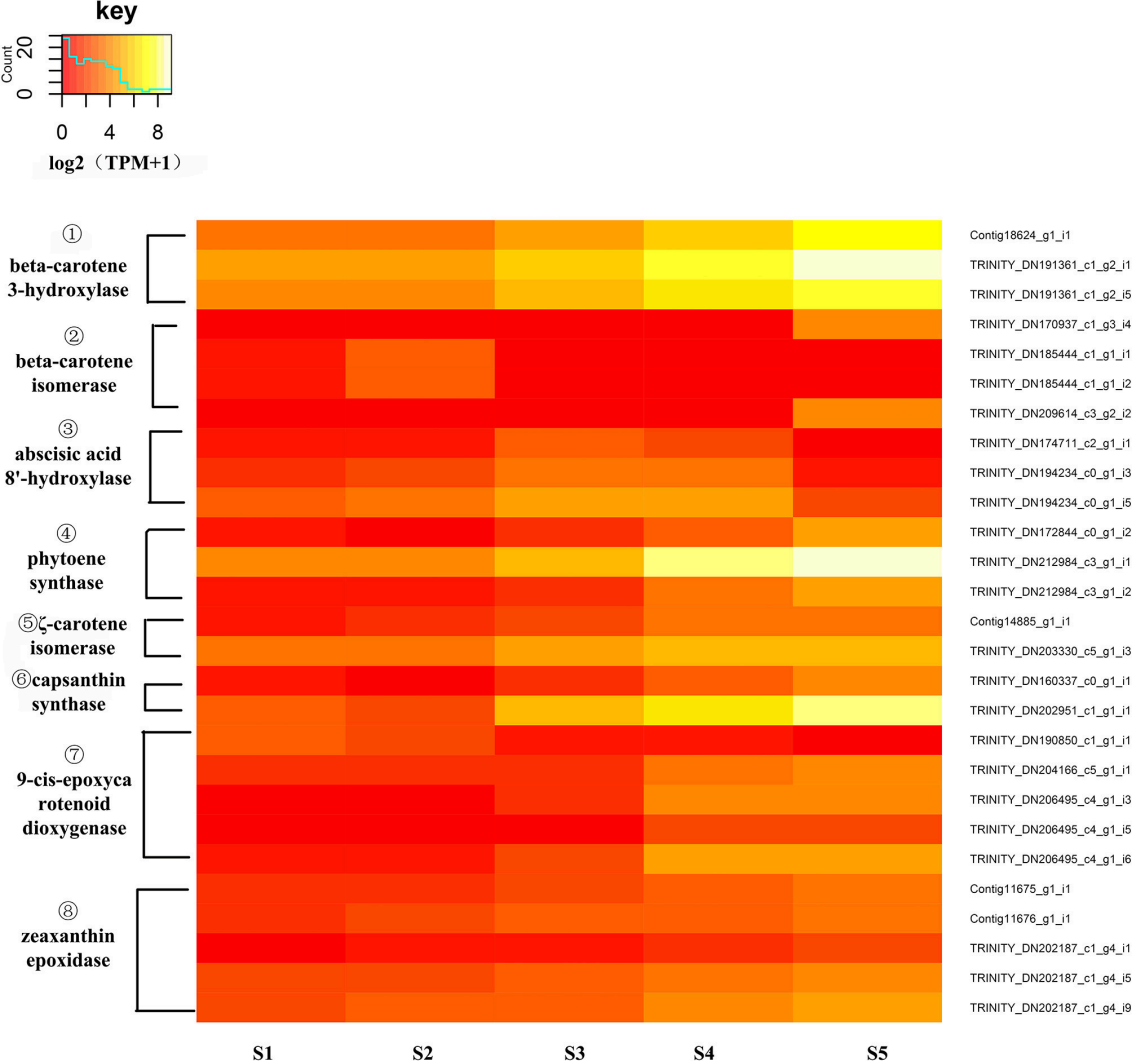
Expression heatmap of differentially expressed genes of carotenoid biosynthesis. The expression of differentially expressed genes (DEGs) displayed as log2 (TMM+1). DEGs genes are defined as genes showing significantly different levels of expression between two stages of flower development. ① CrtZ, β-carotene 3-hydroxylase; ② DWARF27, β-carotene isomerase; ③ ABA8′H, abscisic acid 8′-hydroxylase; ④ PSY, phytoene synthase, ⑤ Z-ISO, ζ-carotene isomerase; ⑥ CCS1, capsanthin synthase; ⑦ NCED, 9-cis-epoxycarotenoid dioxygenase, ⑧ ZEP, zeaxanthin epoxidase.

To identify DEGs of carotenoid biosynthesis, an expression heatmap (Figure 8) was constructed based on the transcriptome. The results indicated that 27 unigenes encoding eight enzymes showed marked changes in expression during the stages of flower development; among these, unigenes encoding PSY, Z-ISO, CrtZ, capsanthin synthase (CCS1), ZEP and 9-cis-epoxycarotenoid dioxygenase(NCED) all showed up-regulated expression during flower development from S1 to S5 while unigenes encoding β-carotene isomerase (DWARF27) and abscisic acid 8'-hydroxylase(ABA8'H) showed different expression pattern to the other DEGs. In the carotenoid pathway, PSY catalyzes the synthesis of phytoene, a precursor of lycopene. The high expression level of *PSY* (Trinity_DN212984_c3_g1_i1) was consistent with this central role in carotenoid biosynthesis. CrtZ is a key enzyme in the biosynthesis of zeaxanthin, a pivotal intermediate of the β branch of the carotenoid pathway, and the high expression level of *CrtZ* appeared consistent with this role. At the same time, the expression levels of two unigenes encoding DWARF27 were down-regulated as flower development progressed. The decreased expression of *DWARF27* might permit a greater flux of β-carotene into the β-carotenoid pathway.

### Analysis of flavonoid pathway gene expression during flower development

To determine the key genes involved in flavonoid biosynthesis, all the unigenes were searched against the flavonoid biosynthesis pathway in the KEGG database. From this, a metabolic map was eventually compiled in which multiple transcripts encoding enzymes of the flavonoid biosynthesis pathway were displayed (Supplementary Figure S6). As indicated on the map, and recognizing that the principal pigments are the flavonols quercetin and kaempferol, it is assumed that flavonoids arise from cinnamoyl-CoA and that the biosynthesis of quercetin occurs through the action of chalcone synthase (CHS, 12 unigenes), chalcone isomerase (CHI, 10 unigenes), naringenin 3-dioxygenase (F3H, 4 unigenes), flavonoid-3′, 5′-hydroxylase (F3′5′H, 6 unigenes), flavonoid 3′-monooxygenase (F3′H, 3 unigenes), and flavonol synthase (FLS, 9 unigenes). In addition to the unigenes cited above, unigenes encoding dihydroflavonol 4-reductase (DFR, 18 unigenes), leucoanthocyanidin reductase (LAR, 10 unigenes), leucoanthocyanidin dioxygenase (LDOX/ANS, 8 unigenes), and anthocyanidin reductase (ANR, 12 unigenes) were identified. It is possible that these genes may be correlated with the biosynthesis of epicatechin or gallocatechin. Additionally, unigenes encoding caffeoyl-CoA *O*-methyltransferase, and shikimate *O*-hydroxycinnamoyltransferase were identified.

**Figure 9:**
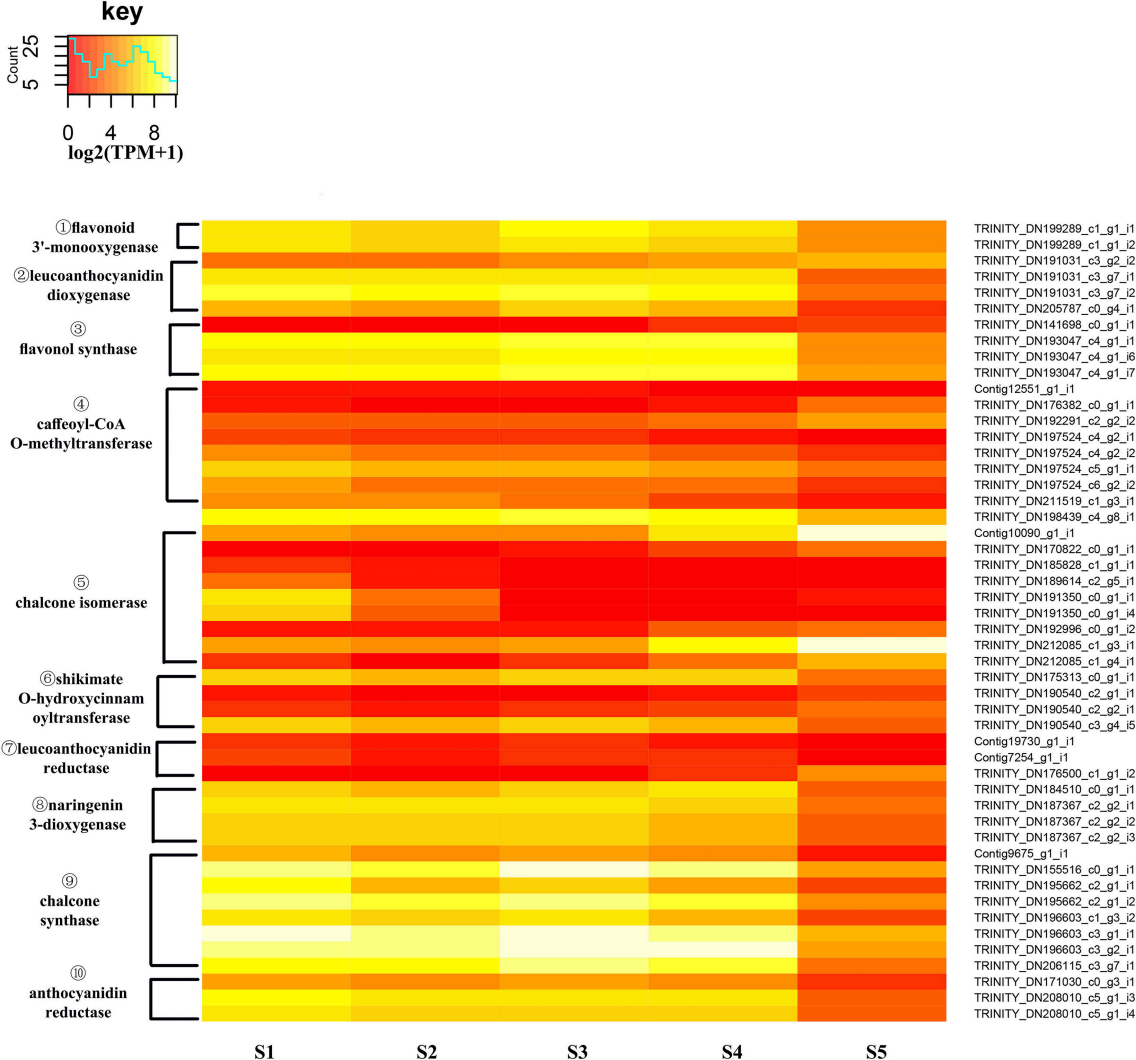
Expression heatmap of differentially expressed genes of flavonoid biosynthesis. The expression of differentially expressed genes (DEGs) displayed as log2 (TMM+1). DEGs genes are defined as genes showing significantly different levels of expression between two stages of flower development. ① F3′H, flavonoid 3′-monooxygenase; ② LDOX/ANS, leucoanthocyanidin dioxygenase; ③ FLS, flavonol synthase; ④ COMT, caffeoyl-CoA *O*-methyltransferase; ⑤ CHI, chalcone isomerase; ⑥ HCT, shikimate *O*-hydroxycinnamoyltransferase; ⑦ LAR, leucoanthocyanidin reductase,; ⑧ F3H, naringenin 3-dioxygenase; ⑨ CHS, chalcone synthase; ⑩ ANR, anthocyanidin reductase.

An expression heatmap was constructed based on the expression of those identified DEGs of the flavonoid pathway (Figure 9). It was found that 49 unigenes, encoding 10 enzymes, showed large changes during flower development. Overall, with the exception of *CHI*s, most of the unigenes showed high expression levels at S1 to S4 and low levels at S5. Among these DEGs, the much higher expression levels of *CHS*s, relative to other unigenes, were suggestive of a particularly important role for *CHS*s in the pathway. FLS catalyzes the formation of flavonols. The high expression levels of *FLS*s observed here are consistent with the essential role of FLS in the biosynthesis of the flavonols quercetin and kaempferol, and with the critical role of *CnFLS1* in yellow pigmentation in *C. nitidissima* (Zhou et al., 2013). High expression levels were also observed for *ANR*s, and these may be related to the accumulation of epicatechin (Supplementary Figure S7). The expression levels of *CHI*s were variable. For four *CHI*s, the highest expression levels were observed to occur in S5 and for two others they were in S1. This result could imply that an elevated level of expression of *CHI* is not necessary for the biosynthesis of flavonols in *C. nitidissima*.

## Discussion

### Transcriptome sequencing and annotation

*Camellia nitidissima* is widely popular, owing to its golden flower color. Flower color is an important trait in many ornamental plants and is regulated through gene expression. An understanding of the molecular mechanisms involved in the generation of the golden color during flower development will assist in the breeding yellow camellias of improved ornamental value. However, scant genomic information is available for *C. nitidissima*. Recently, high-throughput mRNA sequencing(RNA-seq) technology has been widely used to study the transcriptomes of plants because of its reduced cost, high efficiency and rapid output (Wang et al., 2009, 2014; Loraine et al., 2013; Li et al., 2016), particularly for transcriptome profiling in the absence of a reference genome (Grabherr et al., 2011). In the present study, the transcriptomes of golden camellia flowers at different developmental stages were sequenced using the Illumina Hiseq2500 platform and characterized to gain insights into the global regulation of flower pigment metabolism. The transcriptome data reported here will provide a resource for flower development studies in *C. nitidissima* and in other camellias.

In this work, a total of 237,538 of the unigenes from the petals of *C. nitidissima* were annotated by comparison with the public databases Nr, TAIR, Swiss-Prot, and TrEMBL; however, the remaining 229,056 showed no significant matches to any sequences in the public databases, and these numerous newly reported genes specific to *C. nitidissima*, but with unknown functions, will be the subject of future studies.

### Carotenoid accumulation in golden camellia is regulated at the transcriptional level

In *C. nitidissima* flowers, carotenoids play an important role in color formation in the petals (Miyajima et al., 1985; Zhou, 2012). Evidence has indicated that the carotenoid content of petals is mainly controlled at the transcriptional level through the regulation of carotenoid biosynthetic genes (Ohmiya, 2013). In chrysanthemum(*Chrysanthemum morifolium* Ramat.), the expression levels of most carotenoid pathway genes, including *PSY, PDS, ZDS, CRTISO, LCYB, LCYE*, and *CHYB*, increased during petal maturation (Ohmiya et al., 2006). Expression levels of *PSY, ZDS, CRTISO*, and *CHYB* were similarly increased in Asiatic hybrid lily (Yamagishi et al., 2010). In the present study, levels of neoxanthin, violaxanthin, α-carotenoids, and xanthophyll, as determined by HPLC, all increased with the stage of flower development from S1 to S5, and expression levels of *PSY, CrtZ, Z-ISO, CCS1*, and *ZEP* were found to increase concomitantly. Besides, the analysis of co-expression modules showed PSY, *Z-ISO*, ZEP, LUT5 and NCED co-expressed. These results suggest that the expression of these genes plays an important role in carotenoid accumulation in *C. nitidissima* flowers, and further suggests that carotenoid biosynthesis in this system is regulated at the transcriptional level.

### PSY is a key enzyme of carotenoid biosynthesis in golden camellia

In the biosynthesis of carotenoids, PSY is considered to be a rate-limiting enzyme and a key integrator of several signals regulating the carotenoid pathway (Wong et al., 2004; Paine et al., 2005; Giuliano, 2014). Studies indicate that the expression level of *PSY* usually correlates with flower color. In marigold, yellow petals display a lower expression of *PSY* than orange petals (Moehs et al., 2001). In tomato (Giuliano et al., 1993) and in *Gentiana lutea* (Zhu et al., 2002), increased expression levels of *PSY* were observed to occur concomitantly with flower development. The high expression level of *PSY* observed during the stages of flower development in *C. nitidissima* indicates that PSY is a key enzyme of carotenoid biosynthesis.

### High expression level of *FLS, ANS, ANR* regulates the biosynthesis of flavonols and anthocyanidin in golden camellia

Flavonoids are among the most important pigments in the petals of many ornamental plants and produce the widest spectrum of colors, ranging from pale yellow to blue-purple (Zhao and Tao, 2015). A question that remains unclear is why anthocyanins are the principal pigments in the flowers of many red camellia species or cultivars but are not detected in *C. nitidissima* (Lu, 2006; Li et al., 2008; Peng et al., 2011). A possible explanation is that the biosynthesis of anthocyanidins in golden camellia flowers is regulated by one or some genes. In the biosynthesis pathway, *FLS, DFR, ANS*, and *ANR* are the main genes which regulate the biosynthesis of flavonols and anthocyanidin(Tanaka et al., 2008; Mouradov and Spangenberg, 2014). In this research, DEG analysis indicated a high expression level of *FLS, ANS*, and *ANR* while a very low level of *DFR* (Supplementary Table 11). Besides, co-expression network analysis revealed *FLS* and *ANS* co-expressed. These results showed that high expression level of *FLS, ANS* and *ANR* may play important roles in regulating the biosynthesis of flavonols and anthocyanindin.

### Breeding yellow camellias through genetic engineering

Based on the gene expression analysis of the two pathways, and the levels of the pigments, the formation of the golden color may be explained. In the first place, the high expression of carotenoid biosynthetic genes leads to the accumulation of carotenoids in the petals. At the same time, the expression of *FLS, ANS*, and *ANR* regulates the production of flavonols and anthocyanidin. The golden yellow color of the petals is then the result of the combined accumulation of carotenoids and flavonols (as flavonol glucosides).

The basis of color formation in golden camellia points the way to breeding yellow camellias. Yellow camellias may be obtained by increasing the carotenoid content whilst decreasing the synthesis of anthocyanidins. Studies show that regulation of the expression of carotenogenic genes can alter the color of flowers or other organs by promoting the accumulation of carotenoids. Over-expression of *AtPSY* in *Arabidopsis* seedlings increased the carotenoid content and altered the color of seed-derived calli to yellow (Maass et al., 2009). It has been reported that down-regulation of *IbCHY-*β increased total carotenoids in transgenic sweet potato calli and produced a yellow coloration (Kim et al., 2012). In transgenic rice, golden seeds were obtained by introducing and expressing the genes *PSY, crtI*, and *LCYB* (Ye et al., 2000). Up-regulation of *PSY, PDS*, and *LCYB* caused a remarkable increase in the carotenoid content in tomato petals (Giuliano et al., 1993; Corona et al., 1996; Ronen et al., 2000). In *Chrysanthemum morifolium*(Ohmiya et al., 2006) and *Brassica napus*(Zhang et al., 2015), disruption of the expression of *carotenoid cleavage dioxygenase* (*CmCCD4*) resulted in a change in petal color from white to yellow. These studies illustrate the potential to change the flower color of camellias by regulating carotenoid biosynthetic genes, such as *PSY, CHYB*, and others. Regarding the flavonoid pathway, over-expression of *CnFLS1* altered the flower color of *Nicotiana tabacum* to white or light yellow, and metabolic analysis showed a significant increase in flavonols and a reduction of anthocyanins in transgenic plants (Zhou et al., 2013). In transgenic tobacco (*Nicotiana tabacum* cv. Petite Havana SR1), inhibition of the expression of *CHI* and *DFR* led to a loss of anthocyanin (Han et al., 2012). These studies indicate that increasing the content of flavonols and decreasing anthocyanidin synthesis through genetic engineering are feasible. It is thus practicable to breed yellow camellias by targeted regulation of gene expression.

## Conclusions

The transcriptomic analysis presented in this study provides an important foundation for understanding the molecular genetic basis of flower coloration in *C. nitidissima*. The expression of carotenoid biosynthetic genes (such as *PSY, CrtZ*, and *BCH*) leads to the production of carotenoids, whilst at the same time the expression of genes of flavonoid biosynthesis (such as *CHS,F3H, FLS*, and *ANS*) regulate the formation of a second class of pigments, flavonol glucosides. The two types of pigments—carotenoids and flavonol glucosides—function together and cause the petals to be golden yellow. The study reported here points the way toward the breeding of yellow camellias. For breeders, improving the levels of carotenoids and flavonol glucosides and decreasing the synthesis of anthocyanidins through genetic engineering technology should be the focus of future studies.

## Author contributions

XZ was responsible for the data analysis and drafted the manuscript. JC, YZ, XF, and SL prepared cDNA samples for sequencing. YZ and HZ assisted with the data analysis. JL and SN provided helpful comments on the manuscript. YW provided guidance on the whole study and contributed with valuable discussions.

### Conflict of interest statement

The authors declare that the research was conducted in the absence of any commercial or financial relationships that could be construed as a potential conflict of interest.
