# Supplementary material for: De novo Assembly of the Camellia nitidissima Transcriptome Reveals Key Genes of Flower Pigment Biosynthesis
Source: Front Plant Sci. 2017 Sep 7;8:1545. doi: 10.3389/fpls.2017.01545 (PMC5594225; doi:10.3389/fpls.2017.01545)
Supplement: Supplementary file 1 [file Table1.DOCX]

**Supplementary Table 1 Primer sequences used in RT-qPCR**

| **ID** | **Primer** | **5' to 3'** |
| --- | --- | --- |
| Gene 1 | Gene 1—F | GTGGAAGAAACCGTAGGA |
| Gene 1 | Gene 1—R | ATTTGCTCTGTCAGTGGG |
| Gene 2 | Gene 2—F | ATCCAAGTGGGTAAACGA |
| Gene 2 | Gene 2—R | GGACATTCAAGGGACAAG |
| Gene 3 | Gene 3—F | GTTCGGCTCTTCACAATG |
| Gene 3 | Gene 3—R | GAATAACCAGGCACCCAC |
| 18S | 18S—F | GACTCAACACGGGGAAACTTACC |
| 18S | 18S—R | CAGACAAATCGCTCCACCAAC |
