# Supplementary material for: De novo Assembly of the Camellia nitidissima Transcriptome Reveals Key Genes of Flower Pigment Biosynthesis
Source: Front Plant Sci. 2017 Sep 7;8:1545. doi: 10.3389/fpls.2017.01545 (PMC5594225; doi:10.3389/fpls.2017.01545)
Supplement: Supplementary file 2 [file Table2.DOCX]

**Supplementary Table 2 Sample sequencing and quality control**

| **Sample** | **Read length** | **Clean Reads Number** | **Clean Bases Number** | **Raw Q30 Bases Rate (%)** | **Clean Q30 Bases Rate (%)** |
| --- | --- | --- | --- | --- | --- |
| S1_rep1 | 150 | 47,279,536 | 7,091,930,400 | 93.30 | 93.77 |
| S2_rep1 | 150 | 49,653,078 | 7,447,961,700 | 94.31 | 94.69 |
| S3_rep1 | 150 | 48,362,674 | 7,254,401,100 | 94.05 | 94.54 |
| S4_rep1 | 150 | 49,656,662 | 7,448,499,300 | 94.31 | 94.78 |
| S5_rep1 | 150 | 49,206,196 | 7,380,929,400 | 94.36 | 94.75 |
| S1_rep2 | 150 | 49,056,064 | 7,358,409,600 | 94.42 | 94.82 |
| S2_rep2 | 150 | 48,259,180 | 7,238,877,000 | 94.96 | 95.18 |
| S3_rep2 | 150 | 48,386,318 | 7,257,947,700 | 94.16 | 94.61 |
| S4_rep2 | 150 | 49,130,002 | 7,369,500,300 | 94.28 | 94.66 |
| S5_rep2 | 150 | 48,654,066 | 7,298,109,900 | 94.75 | 95.08 |
| S1_rep3 | 150 | 48,658,290 | 7,298,743,500 | 93.50 | 94.06 |
| S2_rep3 | 150 | 50,082,954 | 7,512,443,100 | 94.27 | 94.68 |
| S3_rep3 | 150 | 49,130,946 | 7,369,641,900 | 94.66 | 94.79 |
| S4_rep3 | 150 | 39,120,042 | 5,868,006,300 | 93.25 | 93.61 |
| S5_rep3 | 150 | 43,689,290 | 6,553,393,500 | 93.23 | 93.55 |
