# Supplementary material for: De novo Assembly of the Camellia nitidissima Transcriptome Reveals Key Genes of Flower Pigment Biosynthesis
Source: Front Plant Sci. 2017 Sep 7;8:1545. doi: 10.3389/fpls.2017.01545 (PMC5594225; doi:10.3389/fpls.2017.01545)
Supplement: Supplementary file 3 [file Table3.DOC]

**Supplementary Table 3 Full-length transcript coverage analysis of the assembly result from different platforms**

| **Platform** | **Bridger** | | **SOAP** | | **Trinity** | |
| --- | --- | --- | --- | --- | --- | --- |
| **Total transcripts** | 446526 | | 616740 | | 586523 | |
| **Blast hit** | 110615 | | 46071 | | 181973 | |
| **The distribution of percent length coverage for the top matching database entries** | | | | | | |
| **Hit_pct_cov_bin** | **Count_in_bin** | **>bin_below** | **Count_in_bin** | **>bin_below** | **Count_in_bin** | **>bin_below** |
| **100** | 6395 | 6395 | 4113 | 4113 | 5708 | 5708 |
| **90** | 1980 | 8375 | 1565 | 5678 | 2069 | 7777 |
| **80** | 1433 | 9808 | 1235 | 6913 | 1626 | 10976 |
| **70** | 1395 | 11203 | 1320 | 8233 | 1573 | 12499 |
| **60** | 1339 | 12542 | 1442 | 9675 | 1523 | 12499 |
| **50** | 1406 | 13948 | 1541 | 11216 | 1700 | 14199 |
| **40** | 1552 | 15500 | 1822 | 13038 | 1905 | 16104 |
| **30** | 1930 | 17430 | 2187 | 15225 | 2136 | 18240 |
| **20** | 2036 | 19466 | 2710 | 17935 | 2174 | 20414 |
| **10** | 632 | 20098 | 1592 | 19527 | 608 | 21022 |

**Note:**

Hit_pct_cov_bin: hit percent coverage in the bin

Count_in_bin : Transcripts counts in the bin
