# Supplementary material for: De novo Assembly of the Camellia nitidissima Transcriptome Reveals Key Genes of Flower Pigment Biosynthesis
Source: Front Plant Sci. 2017 Sep 7;8:1545. doi: 10.3389/fpls.2017.01545 (PMC5594225; doi:10.3389/fpls.2017.01545)
Supplement: Supplementary file 4 [file Table4.DOCX]

**Supplementary Table 4 Re-mapping ratio of samples of different assembly platforms**

| Samples ID | Re-mapping ratio | | | Properly paired | | |
| --- | --- | --- | --- | --- | --- | --- |
|  | SOAP | Trinity | Bridger | SOAP | Trinity | Bridger |
| 1-S1 | 99.26% | 99.88% | 99.80% | 80.5% | 86.95% | 82.30% |
| 1-S2 | 99.30% | 99.91% | 99.84% | 82.08% | 88.88% | 84.64% |
| 1-S3 | 99.50% | 99.88% | 99.82% | 86.17% | 90.21% | 85.54% |
| 1-S4 | 99.39% | 99.86% | 99.40% | 82.59% | 88.33% | 85.87% |
| 1-S5 | 99.31% | 99.90% | 99.45% | 82.69% | 89.20% | 87.60% |
| 2-S1 | 99.37% | 99.88% | 99.79% | 82.24% | 88.50% | 84.75% |
| 2-S2 | 99.43% | 99.91% | 99.85% | 84.11% | 89.71% | 85.32% |
| 2-S3 | 99.37% | 99.86% | 99.78% | 82.04% | 88.33% | 84.66% |
| 2-S4 | 99.36% | 99.92% | 99.87% | 81.98% | 88.60% | 84.99% |
| 2-S5 | 99.38% | 99.91% | 99.86% | 83.31% | 89.50% | 85.35% |
| 3-S1 | 99.25% | 99.84% | 99.76% | 80.64% | 87.76% | 82.82% |
| 3-S2 | 99.29% | 99.79% | 99.70% | 80.36% | 87.85% | 83.63% |
| 3-S3 | 99.38% | 99.80% | 99.65% | 82.25% | 89.70% | 85.72% |
| 3-S4 | 99.14% | 99.68% | 99.59% | 81.46% | 88.56% | 84.03% |
| 3-S5 | 99.29% | 99.84% | 99.76% | 83.48% | 89.24% | 84.86% |
| average | 99.33% | 99.86% | 99.73% | 82.39% | 88.75% | 84.81% |
