# Supplementary material for: De novo Assembly of the Camellia nitidissima Transcriptome Reveals Key Genes of Flower Pigment Biosynthesis
Source: Front Plant Sci. 2017 Sep 7;8:1545. doi: 10.3389/fpls.2017.01545 (PMC5594225; doi:10.3389/fpls.2017.01545)
Supplement: Supplementary file 5 [file Table5.DOCX]

| Platform | Complete BUSCOs | Fragment  BUSCOs | Missing  BUSCOs |
| --- | --- | --- | --- |
| Trinity | 80.7% | 11.6% | 7.7% |
| Bridger | 75.1% | 14.3% | 10.6% |
| SOAP | 73.2% | 14.7% | 12.1% |

**Supplmentary Table 5 Completeness assessment by BUSCO software**
