# Supplementary material for: De novo Assembly of the Camellia nitidissima Transcriptome Reveals Key Genes of Flower Pigment Biosynthesis
Source: Front Plant Sci. 2017 Sep 7;8:1545. doi: 10.3389/fpls.2017.01545 (PMC5594225; doi:10.3389/fpls.2017.01545)
Supplement: Supplementary file 6 [file Table6.DOCX]

**Supplementary Table 6 RT-qPCR and RNAseq of three representative genes**

| Gene | Stage | qRT-PCR | | | | RNA-seq | | | |
| --- | --- | --- | --- | --- | --- | --- | --- | --- | --- |
|  |  | rep1 | rep2 | rep3 | average | rep1 | rep2 | rep3 | average |
| gene1 | S1 | 1.300 | 1.428 | 1.291 | 1.340 | 100.346 | 105.892 | 98.120 | 101.453 |
|  | S2 | 0.913 | 0.885 | 0.929 | 0.909 | 81.200 | 78.200 | 89.700 | 83.033 |
|  | S3 | 0.031 | 0.027 | 0.033 | 0.030 | 2.100 | 5.230 | 3.120 | 3.483 |
|  | S4 | 0.051 | 0.057 | 0.049 | 0.052 | 5.321 | 7.120 | 5.000 | 5.814 |
|  | S5 | 0.034 | 0.019 | 0.028 | 0.027 | 3.120 | 4.100 | 3.200 | 3.473 |
| gene2 | S1 | 1.011 | 1.308 | 0.935 | 1.085 | 10.040 | 12.918 | 11.806 | 11.588 |
|  | S2 | 0.877 | 1.122 | 0.815 | 0.938 | 9.568 | 10.371 | 8.146 | 9.362 |
|  | S3 | 1.312 | 1.922 | 1.734 | 1.656 | 9.729 | 8.739 | 10.514 | 9.661 |
|  | S4 | 2.782 | 1.886 | 1.968 | 2.212 | 16.749 | 13.843 | 12.170 | 14.254 |
|  | S5 | 4.345 | 3.832 | 3.940 | 4.039 | 25.609 | 21.731 | 19.965 | 22.435 |
| gene3 | S1 | 2.007 | 2.304 | 2.580 | 2.297 | 8.470 | 10.610 | 9.290 | 9.457 |
|  | S2 | 8.080 | 8.267 | 8.505 | 8.284 | 97.211 | 85.230 | 100.210 | 94.217 |
|  | S3 | 8.974 | 10.015 | 12.188 | 10.392 | 95.124 | 112.120 | 96.210 | 101.151 |
|  | S4 | 13.537 | 15.522 | 14.057 | 14.372 | 113.210 | 135.230 | 129.432 | 125.957 |
|  | S5 | 19.046 | 23.734 | 20.501 | 21.094 | 210.120 | 240.210 | 210.112 | 220.147 |
