# Supplementary material for: De novo Assembly of the Camellia nitidissima Transcriptome Reveals Key Genes of Flower Pigment Biosynthesis
Source: Front Plant Sci. 2017 Sep 7;8:1545. doi: 10.3389/fpls.2017.01545 (PMC5594225; doi:10.3389/fpls.2017.01545)
Supplement: Supplementary file 7 [file Table7.DOCX]

**Supplementary Table 7 COG cluster analysis**

| **Category** | **Functional process** | **Number** | **Percentage** |
| --- | --- | --- | --- |
| A | [A] RNA processing and modification | 4127 | 0.029060106 |
| B | [B] Chromatin structure and dynamics | 1422 | 0.010012956 |
| C | [C] Energy production and conversion | 3970 | 0.027954597 |
| D | [D] Cell cycle control, cell division, chromosome partitioning | 2611 | 0.018385252 |
| E | [E] Amino acid transport and metabolism | 3604 | 0.025377422 |
| F | [F] Nucleotide transport and metabolism | 858 | 0.006041573 |
| G | [G] Carbohydrate transport and metabolism | 6275 | 0.044185162 |
| H | [H] Coenzyme transport and metabolism | 896 | 0.006309148 |
| I | [I] Lipid transport and metabolism | 4408 | 0.031038756 |
| J | [J] Translation, ribosomal structure and biogenesis | 3846 | 0.027081456 |
| K | [K] Transcription | 8339 | 0.058718736 |
| L | [L] Replication, recombination and repair | 2998 | 0.021110297 |
| M | [M] Cell wall/membrane/envelope biogenesis | 2299 | 0.016188317 |
| N | [N] Cell motility | 120 | 0.000844975 |
| O | [O] Posttranslational modification, protein turnover, chaperones | 9557 | 0.067295234 |
| P | [P] Inorganic ion transport and metabolism | 3172 | 0.022335511 |
| Q | [Q] Secondary metabolites biosynthesis, transport and catabolism | 3542 | 0.024940852 |
| R | [R] General function prediction only | 37715 | 0.265568668 |
| S | [S] Function unknown | 10321 | 0.07267491 |
| T | [T] Signal transduction mechanisms | 20990 | 0.147800248 |
| U | [U] Intracellular trafficking, secretion, and vesicular transport | 4490 | 0.031616156 |
| V | [V] Defense mechanisms | 2465 | 0.017357199 |
| W | [W] Extracellular structures | 783 | 0.005513463 |
| Y | [Y] Nuclear structure | 555 | 0.00390801 |
| Z | [Z] Cytoskeleton | 2652 | 0.018673952 |
