# Supplementary material for: De novo Assembly of the Camellia nitidissima Transcriptome Reveals Key Genes of Flower Pigment Biosynthesis
Source: Front Plant Sci. 2017 Sep 7;8:1545. doi: 10.3389/fpls.2017.01545 (PMC5594225; doi:10.3389/fpls.2017.01545)
Supplement: Supplementary file 8 [file Table8.DOCX]

**Supplementary Table 8 Genes differentially expressed between flower development stages**

| **Stage comparison** | **DEGs** | **DEGs (Up)** | **DEGs (Down)** |
| --- | --- | --- | --- |
| S1 vs S2 | 76 | 71 | 5 |
| S1 vs S3 | 1243 | 518 | 725 |
| S1 vs S4 | 2437 | 852 | 1585 |
| S1 vs S5 | 11028 | 5639 | 5389 |
| S2 vs S3 | 236 | 58 | 178 |
| S2 vs S4 | 1065 | 230 | 835 |
| S2 vs S5 | 8066 | 3326 | 4740 |
| S3 vs S4 | 9 | 2 | 7 |
| S3 vs S5 | 4363 | 1964 | 2399 |
| S4 vs S5 | 734 | 402 | 332 |
