# Supplementary material for: De novo Assembly of the Camellia nitidissima Transcriptome Reveals Key Genes of Flower Pigment Biosynthesis
Source: Front Plant Sci. 2017 Sep 7;8:1545. doi: 10.3389/fpls.2017.01545 (PMC5594225; doi:10.3389/fpls.2017.01545)
Supplement: Supplementary file 9 [file Table9.DOCX]

**Supplementary Table 9 Correlation of filtered modules with traits**

| **Trait** | **Quercetin** | **Kaempferol** | **Violaxanthin** | **Xanthophyll** | **Neoxanthin** | **α-Carotene** | **Diameter** |
| --- | --- | --- | --- | --- | --- | --- | --- |
| **Related module** | M49  M50  M53  M54  M55  M57 | M1  M19  M24  M38  M67 | M1  M31  M33  M46  M54  M55  M65 | M1  M3  M31  M33  M54  M55 | M1  M3  M31  M33  M46  M54  M55  M65 | M3  M4  M33  M54  M55 | M3  M4  M11  M13  M54  M55 |
