# Supplementary material for: De novo Assembly of the Camellia nitidissima Transcriptome Reveals Key Genes of Flower Pigment Biosynthesis
Source: Front Plant Sci. 2017 Sep 7;8:1545. doi: 10.3389/fpls.2017.01545 (PMC5594225; doi:10.3389/fpls.2017.01545)
Supplement: Supplementary file 10 [file Table10.DOCX]

**Supplementary Table 10 Expression levels of *DFRs***

| **Number** | **Gene name** | **Stage1** | **Stage2** | **Stage3** | **Stage4** | **Stage5** |
| --- | --- | --- | --- | --- | --- | --- |
| 1 | *DFR* | 11.35 | 6.75 | 11.41 | 9.81 | 3.85 |
| 2 | *DFR* | 1.79 | 2.12 | 0.89 | 0.99 | 1.13 |
| 3 | *DFR* | 24.27 | 20.94 | 7.72 | 4.61 | 9.07 |
| 4 | *DFR* | 0.00 | 0.00 | 0.19 | 0.07 | 0.00 |
| 5 | *DFR* | 7.78 | 3.97 | 7.15 | 6.26 | 3.28 |
| 6 | *DFR* | 1.00 | 0.86 | 0.63 | 1.89 | 0.55 |
| 7 | *DFR* | 1.75 | 1.07 | 0.51 | 0.79 | 0.41 |
| 8 | *DFR* | 1.94 | 1.81 | 0.89 | 1.35 | 1.13 |
| 9 | *DFR* | 0.42 | 0.00 | 0.00 | 0.00 | 0.00 |
| 10 | *DFR* | 0.05 | 0.3 | 0.27 | 0.00 | 0.14 |
| 11 | *DFR* | 0.65 | 0.77 | 0.80 | 0.56 | 0.74 |
| 12 | *DFR* | 2.93 | 3.26 | 2.51 | 1.891 | 3.08 |
| 13 | *DFR* | 0.46 | 0.99 | 0.99 | 0.48 | 0.91 |
| 14 | *DFR* | 4.28 | 4.03 | 3.65 | 2.76 | 2.64 |
| 15 | *DFR* | 0.18 | 0.00 | 0.00 | 0.00 | 0.00 |
| 16 | *DFR* | 25.12 | 24.81 | 16.87 | 4.07 | 0.75 |
| 17 | *DFR* | 130.87 | 68.82 | 80.36 | 63.25 | 49.10 |
| 18 | *DFR* | 68.12 | 41.70 | 46.04 | 38.75 | 22.45 |
