# Supplementary figures and images for: De novo Assembly of the Camellia nitidissima Transcriptome Reveals Key Genes of Flower Pigment Biosynthesis
Source: Front Plant Sci. 2017 Sep 7;8:1545. doi: 10.3389/fpls.2017.01545 (PMC5594225; doi:10.3389/fpls.2017.01545)

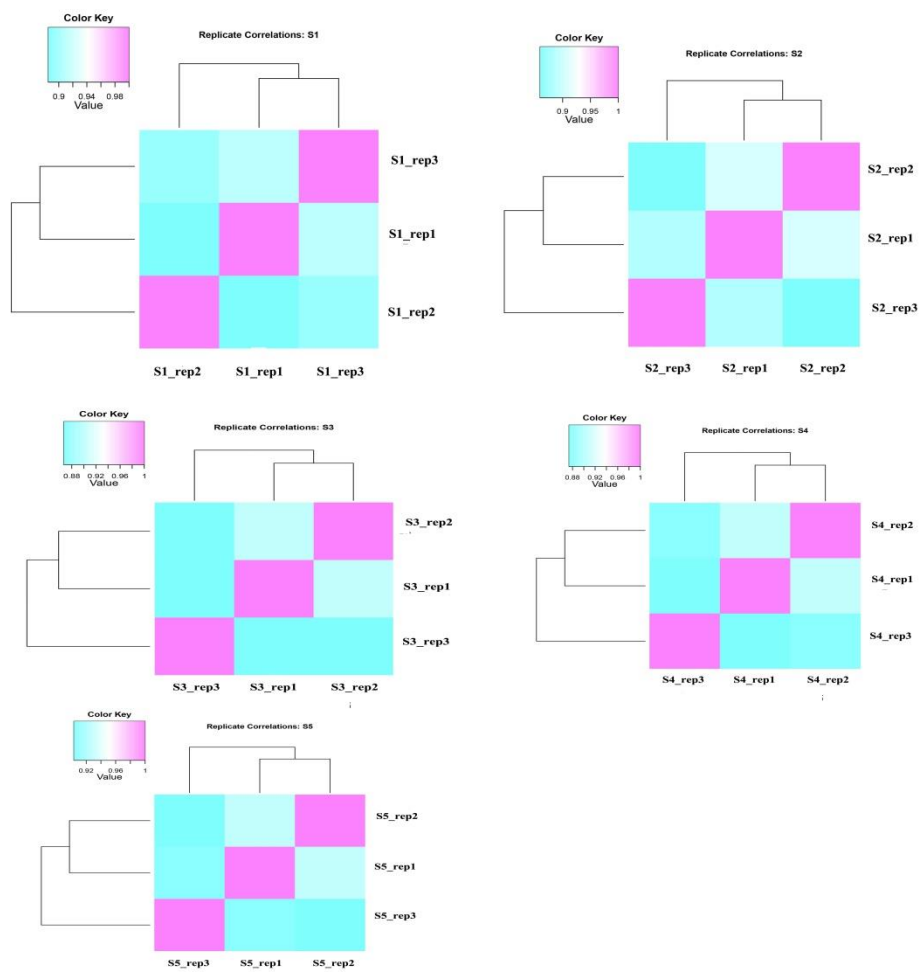

**Supplementary Figure S1. Correlation analysis of biological replicates**

Supplement: Supplementary file 12 [file Image1.PDF]

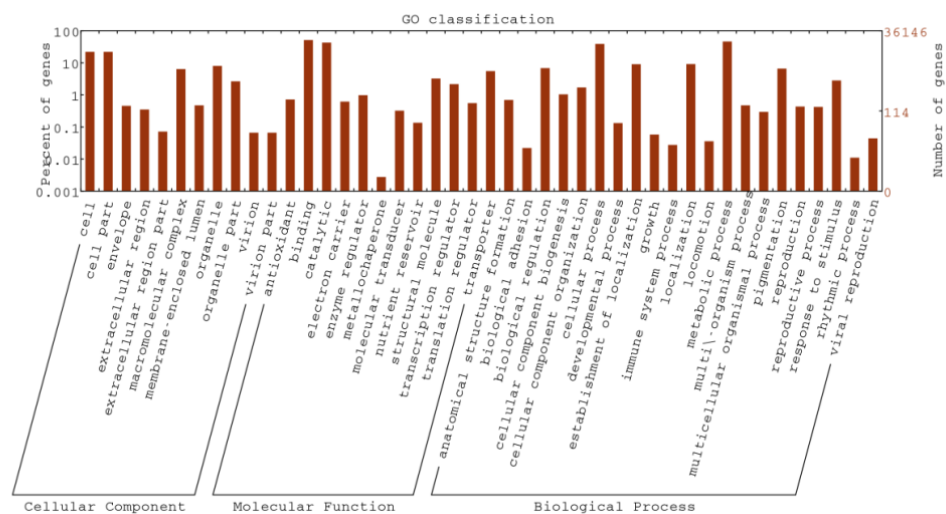

Supplementary Figure S2. Gene ontology classification analysis of *Camellia nitidissima*

Supplement: Supplementary file 13 [file Image2.PDF]

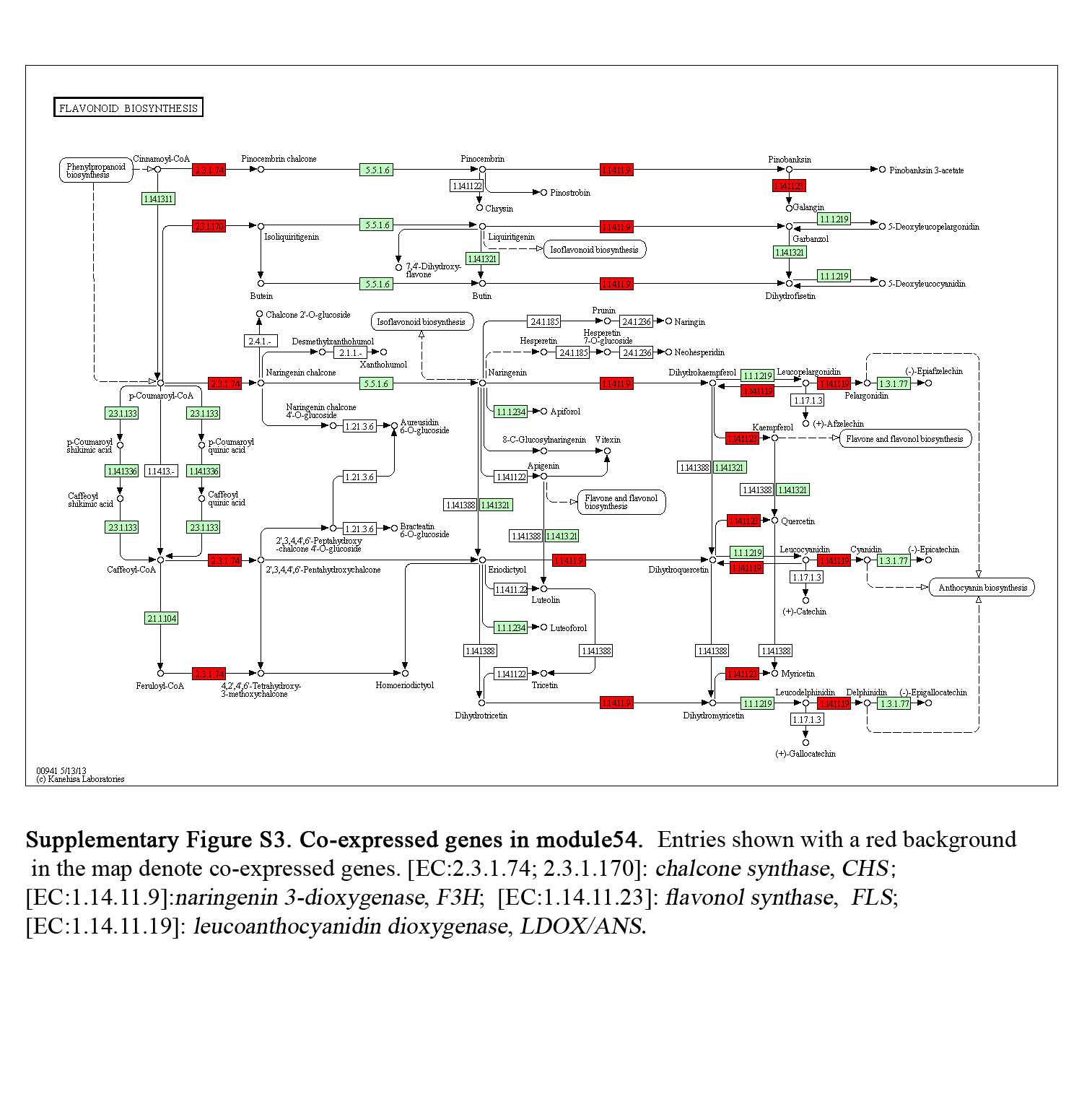

Supplement: Supplementary file 14 [file Image3.JPEG]

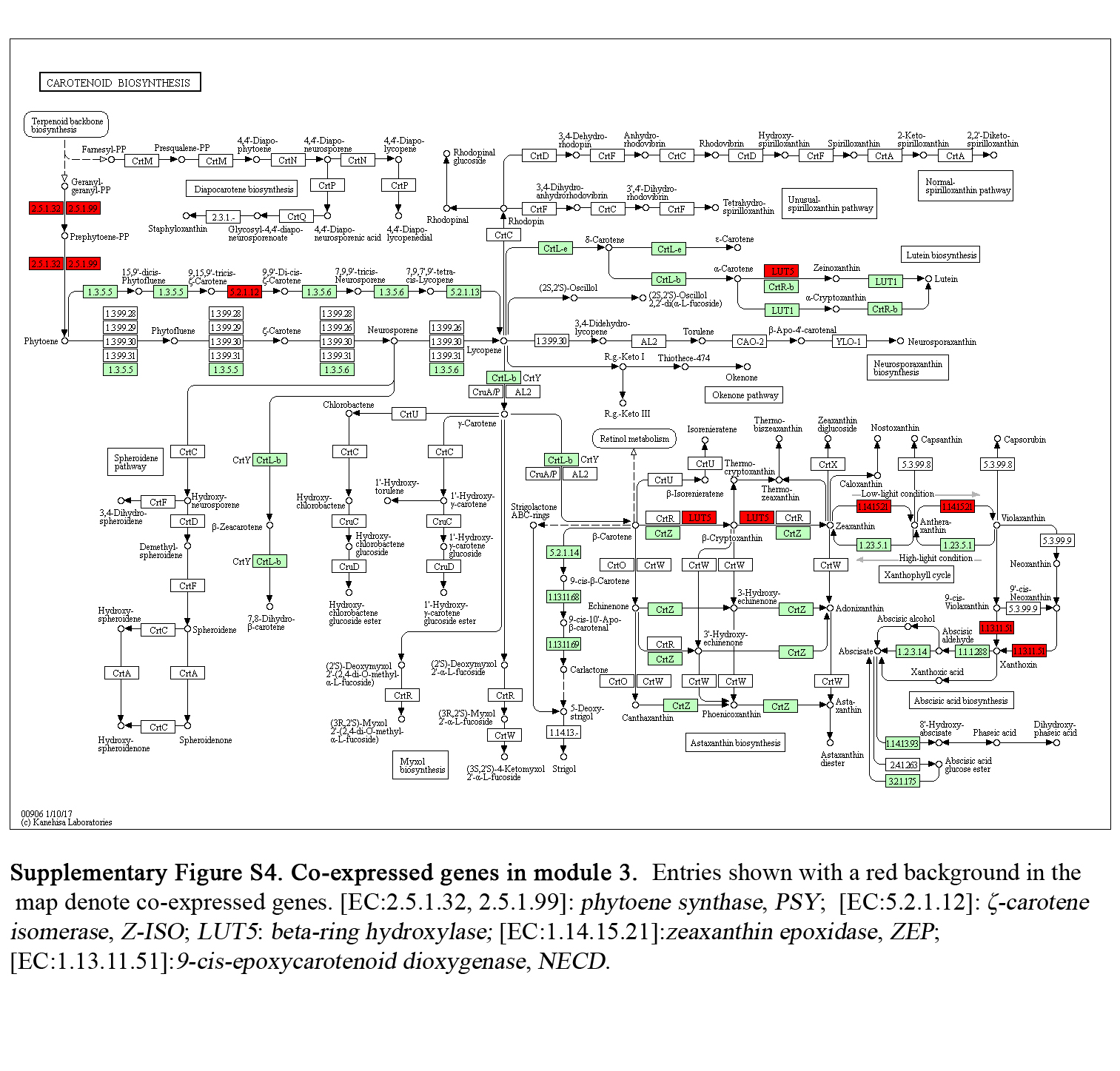

Supplement: Supplementary file 15 [file Image4.JPEG]

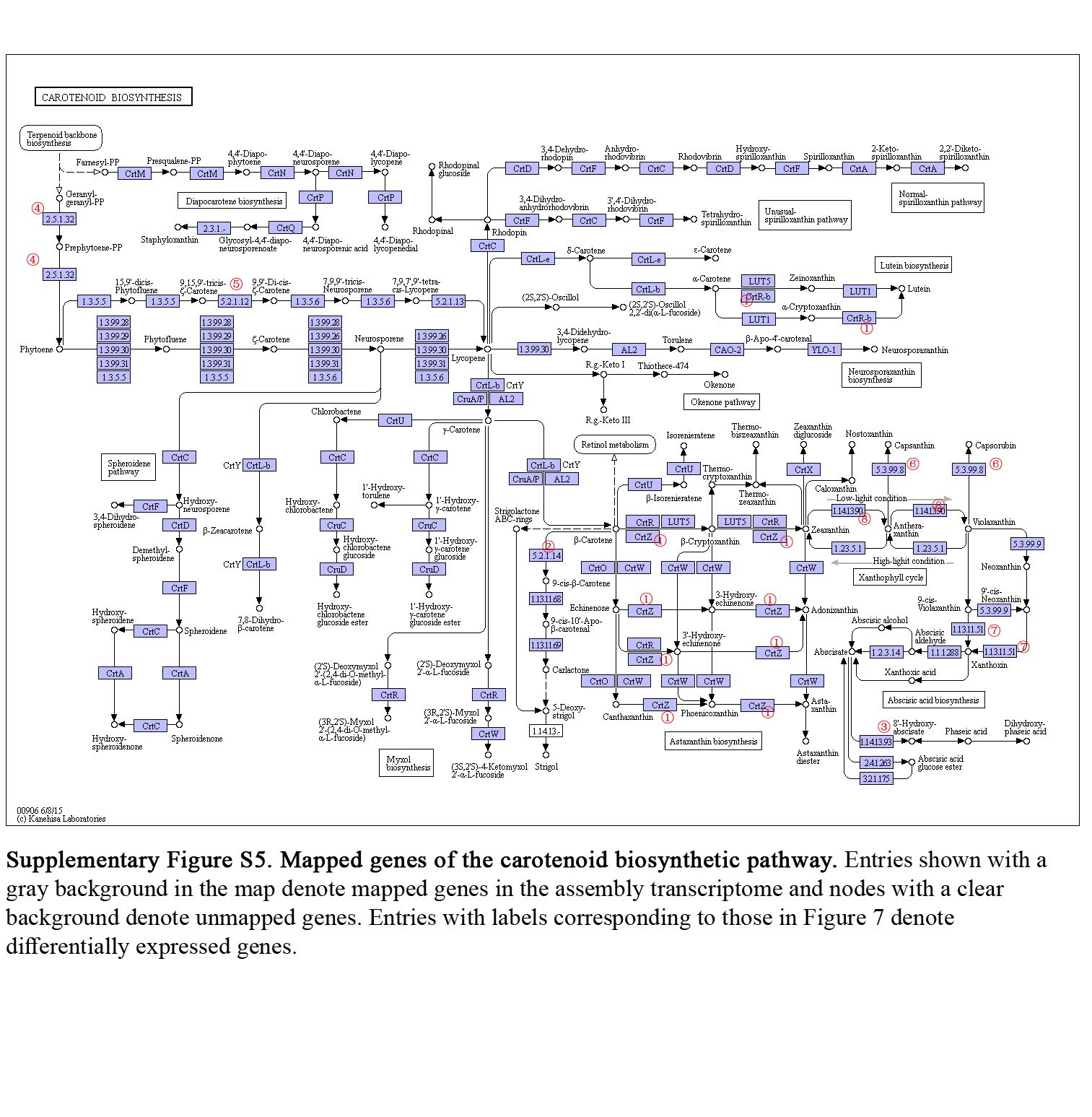

Supplement: Supplementary file 16 [file Image5.JPEG]

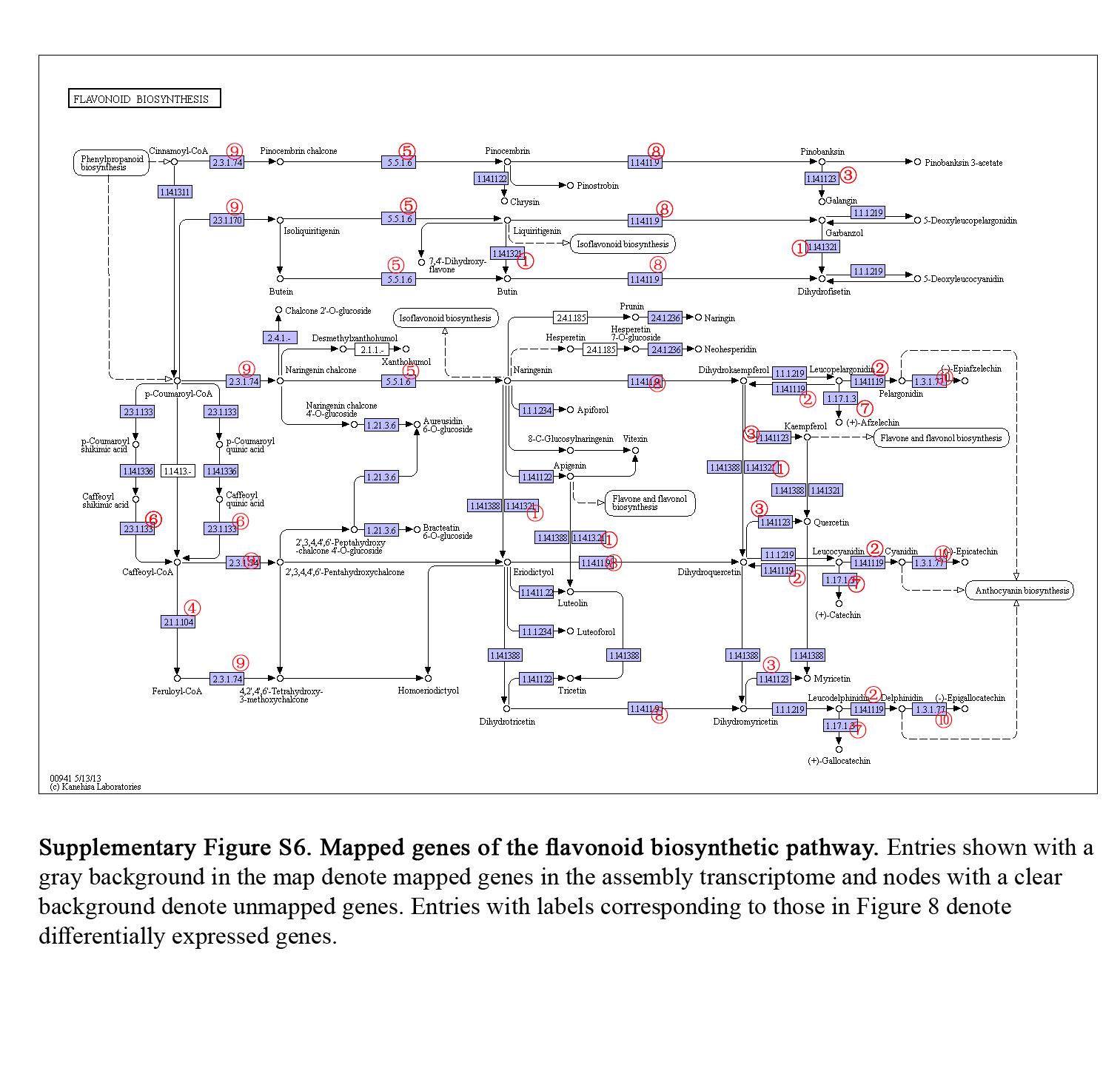

Supplement: Supplementary file 17 [file Image6.JPEG]

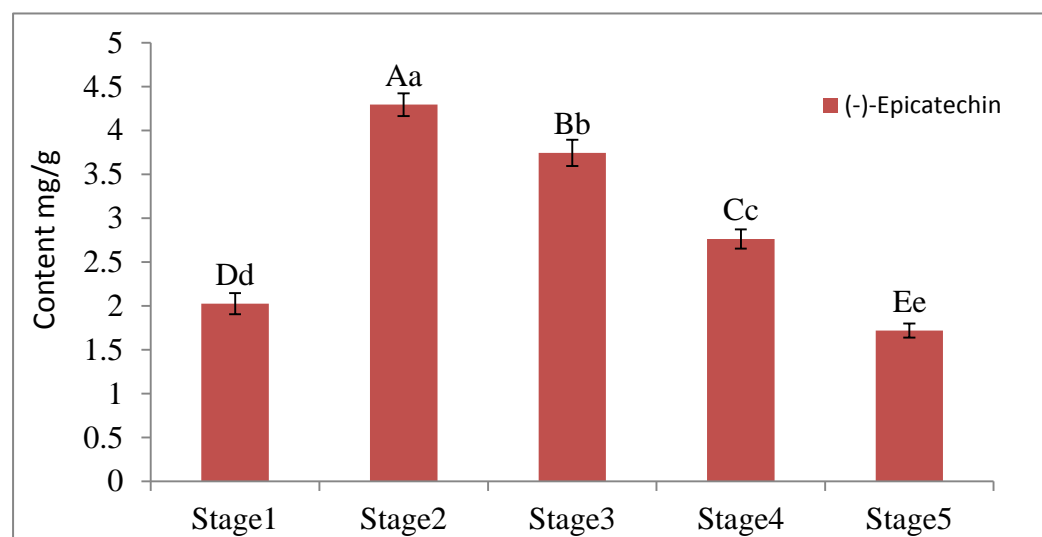

**Supplementary Figure S7. Epicatechin analysis during floral development stages**

Supplement: Supplementary file 18 [file Image7.PDF]
